# Identification of annotated bioactive molecules that impair motility of the blood fluke *Schistosoma mansoni*

**DOI:** 10.1016/j.ijpddr.2020.05.002

**Published:** 2020-06-01

**Authors:** Thomas B. Duguet, Anastasia Glebov, Asimah Hussain, Shashank Kulkarni, Igor Mochalkin, Timothy G. Geary, Mohammed Rashid, Thomas Spangenberg, Paula Ribeiro

**Affiliations:** a Institute of Parasitology, McGill University, Sainte-Anne-de-Bellevue, Quebec, Canada; b EMD Serono Research and Development Institute, Billerica, MA, USA; c Global Health Institute of Merck, Ares Trading S.A., a subsidiary of Merck KGaA (Darmstadt, Germany), Eysins, Switzerland

**Keywords:** Schistosomiasis, Anthelmintic, Screening, Motility, NPS-2143, GPCR

## Abstract

Neglected tropical diseases are of growing worldwide concern and schistosomiasis, caused by parasitic flatworms, continues to be a major threat with more than 200 million people requiring preventive treatment. As praziquantel (PZQ) remains the treatment of choice, an urgent need for alternative treatments motivates research to identify new lead compounds that would complement PZQ by filling the therapeutic gaps associated with this treatment. Because impairing parasite neurotransmission remains a core strategy for control of parasitic helminths, we screened a library of 708 compounds with validated biological activity in humans on the blood fluke *Schistosoma mansoni*, measuring their effect on the motility on schistosomulae and adult worms. The primary phenotypic screen performed on schistosomulae identified 70 compounds that induced changes in viability and/or motility. Screening different concentrations and incubation times identified molecules with fast onset of activity on both life stages at low concentration (1 μM). To complement this study, similar assays were performed with chemical analogs of the cholinomimetic drug arecoline and the calcilytic molecule NPS-2143, two compounds that rapidly inhibited schistosome motility; 17 arecoline and 302 NPS-2143 analogs were tested to enlarge the pool of schistosomicidal molecules. Finally, validated hit compounds were tested on three functionally-validated neuroregulatory *S. mansoni* G-protein coupled receptors (GPCRs): Sm5HTR (serotonin-sensitive), SmGPR2 (histamine) and SmD2 (dopamine), revealing NPS-2143 and analogs as potent inhibitors of dopamine/epinine responses on both human and *S. mansoni* GPCRs. This study highlights the potential for repurposing known human therapeutic agents for potential schistosomicidal effects and expands the list of hits for further progression.

## Highlights

• Screening annotated bioactive molecules to ease new treatment search.
• Coupling motility recording of treated parasite and molecular target search.
• Exploiting chemical analogs to expand bioactive compound search.
• The larvicidal NPS-2143 acts on dopaminergic GPCRs.

## Graphical abstract

**Figure:**
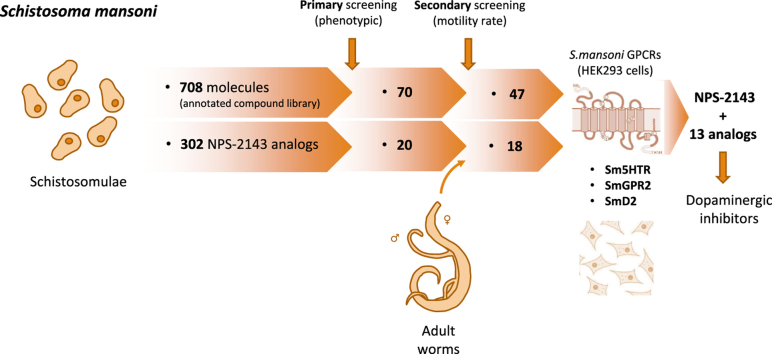

## 1 Introduction

Schistosomiasis is a life-threatening neglected tropical disease caused by parasitic trematodes in the genus *Schistosoma*, in particular *S. mansoni*, *S. haematobium* and *S. japonicum*, which account for the vast majority of human infections (Colley et al., 2014). Ranking amongst the most important parasitic disease, schistosomiasis affects more than 200 million people, 90% of whom live in sub-Saharan Africa (Colley et al., 2014; Lai et al., 2015).

Currently approved therapies for schistosomiasis control include oxamniquine (Foster et al., 1973; Foster, 1987), which has limited action on *S. japonicum* and *S. haematobium* (Cioli et al., 2014). In contrast, praziquantel (PZQ), introduced in the 1970s (Gonnert and Andrews, 1977), has broad-spectrum activity on schistosome species (and other flatworms), but has limited activity against juvenile stages of the parasite. Over time, drug resistance may become a major issue, as reduced PZQ susceptibility has been demonstrated in the laboratory as well as in field isolates of *S. mansoni* (Melman et al., 2009; Mader et al., 2018). Finally, unlike oxamniquine, which impairs nucleic acid metabolism after activation by a sulfotransferase enzyme (Valentim et al., 2013), our understanding of the mechanism of action of PZQ is limited, which hinders rational drug discovery paradigms to identify alternative or complimentary control strategies aimed at PZQ-related pathways (Aragon et al., 2009; Salvador-Recatala and Greenberg, 2012).

Consequently, novel broad-spectrum anthelmintics that target both adult and juvenile human schistosome species would be a vast improvement for the treatment and prevention of schistosomiasis. To support the need to efficiently target the parasite over the span of its long development cycle, including persistent juvenile forms in the host, a longer half-life would be an advantage for a new drug.

Massive efforts are therefore needed to identify novel molecules that can meet the aforementioned criteria. For nematodes, the anthelmintic families of macrocyclic lactones, imidazothiazoles and aminoacetonitrile derivatives, which target the nervous system of multiple species of plant and animal parasites, result in dramatic and rapid worm burden reductions (Wolstenholme, 2012, Holden-dye and Walker, 2014). For schistosomes, motility remains an essential function underlying the continuity of the parasite life-cycle, from skin penetration by cercariae to bloodstream navigation of schistosomulae and site-holding by adult worms. As for neuromodulatory anthelmintics, a pharmacological treatment interfering with motility would eliminate the parasite and/or disrupt the process of infection. Complementing phenotypic screening, current research seeks to identify potentially targetable proteins for mechanism-based drug discovery programs, most of which are ligand-gated ion-channels, G-protein coupled receptors (GPCRs) and other key proteins involved in neuromuscular signalling (Hamdan et al., 2002a; Taman and Ribeiro, 2009; El-Shehabi and Ribeiro, 2010; El-Shehabi et al., 2012; MacDonald et al., 2014; Patocka et al., 2014; El-Sakkary et al., 2018).

Despite technical limitations imposed by the challenge of maintaining the parasite life-cycle, a number of schistosome assays/methods have been proposed with the aim of improving compound screening (Abdulla et al., 2009; Paveley et al., 2012; Asarnow et al., 2015; Panic et al., 2015a; Lombardo et al., 2019). These methods led to the identification of molecules with promising activity, such as neuromodulatory compounds that impair the tyrosine-derivative signaling system (El-Sakkary et al., 2018). Among them, a high-throughput screen (HTS) of 300,000 molecules recently identified seven promising lead compounds that affect larval, juvenile and adult motility (Mansour et al., 2016). Other mechanism-based methods have screened compounds against strategic molecular targets, including the *S. mansoni* serotoninergic GPCR Sm5HTR expressed in HEK293 cells (Chan et al., 2016). Indeed, considering the proposed role of flatworm serotoninergic and dopaminergic neurons in PZQ activity (Chan et al., 2014), a limited screen of Sm5HTR ligands demonstrated the relevance of using *S. mansoni* GPCRs as antiparasitic targets. Such an approach echoes the recent low throughput screening of 28 drugs that modulate the signaling systems of schistosomes, some of them acting on dopamine and octopamine-sensitive receptors (El-Sakkary et al., 2018). Similarly, the adult tegumental *S. mansoni* NAD^+^ catabolizing enzyme (SmNACE) was proposed as a key enzyme impacting NAD^+^-dependent pathways of the human immune system (Kuhn et al., 2010). To this end, a yeast-based HTS of 14,300 molecules identified two anthocyanidins as potent SmNACE inhibitors. Another well-characterized *S. mansoni* druggable target, a thioredoxin glutathione reductase (Eweas and Allam, 2018), was used in a target-based HTS of 59,360 compounds to identify inhibitors, which revealed three molecules that killed schistosomulae and adults (Li et al., 2015).

These methods highlight the need to explore a broader range of annotated bioactive molecules with potential antischistosomal activity. We analyzed a customized library of 708 tool compounds with validated human biological and pharmacological activities (Selleck Chemicals LLC, Houston, TX), including the nervous system. Exposure of schistosomula and adult stages identified 70 molecules in this collection that induce distinct phenotypes or mortality of schistosomulae, adults or both. Hits with strong activity against larval and adult stages were then further tested against key *S. mansoni* neuromodulatory class A GPCRs previously characterized in our laboratory with the aim of validating potential targets (Taman and Ribeiro, 2009; El-Shehabi and Ribeiro, 2010; Patocka et al., 2014).

Along with other drug repurposing initiatives (Ramamoorthi et al., 2015), this approach leverages annotated bioactive molecules that show crossover activity against schistosomes to potentially lead to new target-based drug discovery programmes.

## 2 Materials and methods

### 2.1 Parasites

Puerto Rican strain *Biomphalaria glabrata* snails infected with *S. mansoni* were kindly provided by Dr. Fred Lewis (Biomedical Research Institute and BEI Resources, MD, USA). Schistosomulae were obtained by exposing six to eight weeks-old snails to continuous light for 2 h at 32 °C. The resulting suspension of cercaria was then mechanically transformed as described (Lewis, 2001) and cultured in Opti-MEM/antibiotics (Thermo Fisher Scientific, Waltham, MA) containing 100 μg/mL streptomycin and 100 units/ml penicillin supplemented with 5% fetal bovine serum (FBS; Thermo Fisher). Parasite cultures were incubated at 37 °C with 5% CO_2_ for at least 10 days with no apparent loss of viability. At this point, animals with internal dark granulations and with no observable body contraction/relaxation cycles were considered dead.

Adult parasites were collected from 40 day-old female CD1 mice (Charles River) previously inoculated by the tail infection method (Lewis, 2001). Six weeks post-infection, mice were sacrificed, and adult worms extracted after perfusion of in the hepatic portal vein and mesenteric venules (Lewis, 2001). Animal procedures were reviewed and approved by the Facility Animal Care Committee of McGill University (Protocol No. 3346) and were conducted in accordance with the guidelines of the Canadian Council on Animal Care.

### 2.2 Chemical library

A structurally diverse collection of 708 compounds targeting major cell signaling pathways was acquired from Selleck Chemicals LLC (Houston, TX) (Sup. Table 1). A large subset of the screening library was subclassified as modulators of ligand-gated ion-channels (L2700; 52 compounds), inhibitors of GPCRs (L2200; 254 compounds) and target-selective inhibitors (L3500; 464 compounds). Names, chemical structures, human protein targets and physiological pathways are provided for each compound in Supplementary Materials. Compounds were dissolved in dimethyl sulfoxide (DMSO) at a concentration of 10 mM and stored at -20 °C. Derivatives of some active compounds were provided by Merck KGaA (Darmstadt, Germany) and maintained in identical conditions.

### 2.3 Phenotypic primary screen assay

Three-day-old schistosomulae were placed in individual wells of a 48-well plate (200 animals/well) and maintained in 300 μL OPTI-MEM (Gibco, Thermo Fisher Scientific, Carlsbad, CA) supplemented with 5% dialyzed FBS (Gibco) without antibiotics as previously described (MacDonald et al., 2015). After 15 min incubation at room temperature, animals were exposed to each of the 708 compounds at a final concentration of 10 μM for 24 h at 37 °C, 5% CO_2_. Schistosomulae were monitored under a stereo microscope (SMZ1270, Nikon). Phenotypic observations were made by analyzing schistosomulae, 24 h after drug addition to assess any immediate effect and five days post-compound exposure to observe long-term impacts on both the movement and overall body shape in comparison to control animals exposed to DMSO only. Viability was calculated five days post-compound addition with the methylene blue dye exclusion assay (Gold, 1997).

### 2.4 Motility assay

Schistosomulae (200/well) and adult parasites (unpaired worms) were maintained in 300 μL OPTI-MEM with 5% dialyzed FBS (without antibiotics) and exposed to compounds at 1 and 10 μM for 24 h. A kinetic assay consisting of 1, 3, 6 and 12 h incubation times was performed on selected hits by motility monitoring using a stereo microscope (SMZ1270, Nikon) for adults and an inverted microscope for schistosomulae (eclipse TE2000-U, Nikon) connected to a monochrome camera (DS-Qi2, Nikon) for image acquisition. Images were recorded at a rate of 10 frames/s for a period of 1 min and videos were analyzed using ImageJ software (version 1.41, NIH, USA). Parasite movement quantification and data analysis protocols were applied using the pixel displacement method (Patocka et al., 2014; Rashid et al., 2015) and compared to control parasites treated with DMSO. Parasites were then washed several times in Opti-MEM, 5% FBS and incubated up to an additional three days (schistosomulae) or five days (adult worms) at 37 °C, 5% CO_2_ and motility was recorded as described above.

### 2.5 Cell culture and G-coupled protein receptor expression

Two HEK293 strains stably expressing the *S. mansoni* GPCR sequences Sm5HTR (Patocka et al., 2014; Rashid et al., 2015) and SmD2 (Taman and Ribeiro, 2009), were available in our laboratory and receptor expression was validated by immunofluorescence according to the authors’ instructions. The SmGPR2 sequence previously identified (El-Shehabi and Ribeiro, 2010) was codon optimized for mammalian expression and subcloned in the vector pCI-neo (Addgene, USA) using the *Xba*I and *Not*I restriction enzymes. A FLAG fusion tag (DYKDDDDK) was introduced at the N-terminal end of the receptor sequence. Stable transfection of HEK293 cells (ATCC; CRL-1573) was performed using the X-tremeGENE 9 DNA transfection reagent (Sigma Aldrich). Cells were then cultured at 37 °C, 5% CO_2_ in Opti-MEM supplemented with 5% FBS and maintained in 400 μg/mL G418 (Sigma Aldrich) for selection of transformants. As described elsewhere (Patocka and Ribeiro, 2007), SmGPR2 expression was validated by immunofluorescence using an anti-FLAG primary antibody (Sigma) and subsequently FITC-conjugated anti-mouse IgG. All cell lines expressing *S. mansoni* GPCRs were then transfected with the GloSensor encoding plasmid p22F (Promega) and media was supplemented with 100 μg/mL hygromycin for stable expression (ThermoFisher). The p22F function was verified by measuring relative luminescence intensity (RLU) in the presence of 20 μM forskolin (Sigma Aldrich).

### 2.6 cAMP luminescence assays

Adherent HEK293 cells were seeded at a density of 5 x 10^4^ cells/well (96-well plate) in Opti-MEM without FBS or antibiotics and incubated overnight at 37 °C and 5% CO_2_ prior to the assay. In place of the media, 100 μL HBSS supplemented with 0.1% bovine serum albumin (BSA), 500 μM 3-isobutyl-1-methylxanthine (IBMX, Sigma Aldrich), 20 mM HEPES (pH 7.4) and 1 mg/mL D-luciferin (Goldbio) was added to each well. After a 2 h incubation period at room temperature, luminescence background was recorded, and compounds and control ligands were added at 10X concentration. Immediately after compound addition, luminescence was read over a 45 min period to detect compounds stimulating cAMP release through receptor activation. If no increase of luminescence was observed, the addition of a submaximal concentration (50 μM) of the receptor ligand (*i.e*., serotonin, histamine or epinine) was performed to force receptor activation and identify inhibitory activity of compounds over a subsequent 45 min reading time. For each assay, luminescence responses were recorded in triplicate and repeated with four independant cell batches. Compounds were also added to naïve Glo HEK293 cells (no receptor) and the robustness of the assay was calculated as:

$$Z^{'}=\;1-\frac{3\times \left(SD_{max}+SD_{min}\right)}{Mean_{max}-Mean_{min}}$$

with max and min referring respectively to cells expressing GPCRs and Glo (no receptor) cells. Cellular viability was assessed using a CellTiter Glo 2.0 kit (Promega). Glo cells were exposed to 10 μM of each hit compound for 3 h in Opti-MEM at 37 °C, 5% CO_2_. An equimolar concentration of tamoxifen (Sigma Aldrich) was used as a positive control. 2X cell lysis media was then added and end-point luminescence recorded after a 10 min stabilization time.

### 2.7 Statistical analysis

Statistical tests were performed using GraphPad Prism version 7.0c software (San Diego, CA, USA). Data are presented as mean ± standard error of the mean (SEM). Motility data were analyzed with Student's t-test with p < 0.05 cut-off.

### 2.8 Compound analysis

Active compounds (hits) from the Selleckchem library were analyzed using TIBCO Spotfire version 7.11 software (Palo Alto, CA, USA). Physicochemical properties of the hits were calculated using ChemDraw version 17.1 software (PerkinElmer, Inc.).

## 3 Results

### 3.1 A primary screen of a customized compound library identified 70 out of 708 molecules impairing viability of *S. mansoni* schistosomulae and/or inducing observable phenotypes

**Fig. 1:**
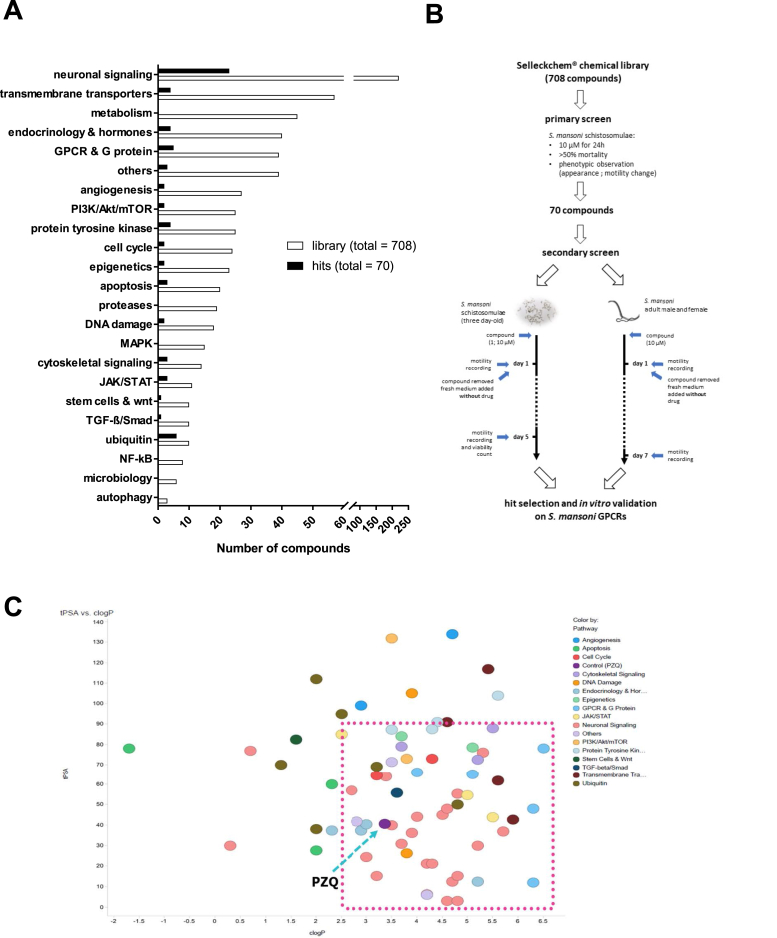
**Compound screening workflow from a customized Selleckchem**® **drug library.** (A) Distribution of the whole compound library (top pie chart) and the primarily selected compounds (bottom pie chart) per reported functional pathway. (B) Stepwise schematic of compound screening and hit selection based on phenotypic observation and motility recording of compound-treated *S. mansoni* schistosomulae and adult stages (C) Compound analysis in Spotfire: Plot of tPSA vs cLogP of compounds screened from Selleckchem® library.

The Selleckchem® chemical library contains a wide range of small molecules with known therapeutic indications and biological and pharmacological activities in humans. The customized primary collection of 708 compounds (Sup. Table 1), sub-classified according to the targeted signaling pathway (Fig. 1A), was assayed. Among them, 31% are inhibitors, agonists or antagonists of ligand-gated ion-channels and GPCRs involved in neuronal signaling (Fig. 1A, Sup. Table 1). The second most represented group (8%) includes inhibitors of the transport of Ca^2+^, K^+^ and Na^+^ ions. Other compounds were classified in subsets of inhibitors targeting a variety of cellular signaling pathways through the inhibition of non-neuronal GPCRs and other receptors related to metabolism or hormone regulation cycles (Fig. 1A, Sup. Table 1).

A primary screen of the whole collection was performed by exposing *S. mansoni* schistosomulae to compounds at 10 μM for 24 h. Visual observation of motility change and viability tests were performed to assess effects. As a result, 70 molecules out of 708 were selected (9.8% hit rate) for further investigation (Fig. 1A and B, Sup. Table 2), among which 31.4% were neuronal signaling-related compounds (Fig. 1A). 35.7% of the compounds reduced viability ≥50%, with nine killing all parasites by four days post-exposure. Notably, the six of the 10 initial compounds that act in ubiquitin-related pathways induced high mortality (45.5–100%). The physicochemical properties of the screened compounds were calculated and a plot of tPSA against cLogP showed that 70% of the hits have a cLogP value of >2.5 and tPSA of <90 Å^2^, including praziquantel (PZQ, cLogP 3.3; tPSA 90 Å^2^) (Fig. 1C).

**Fig. 2:**
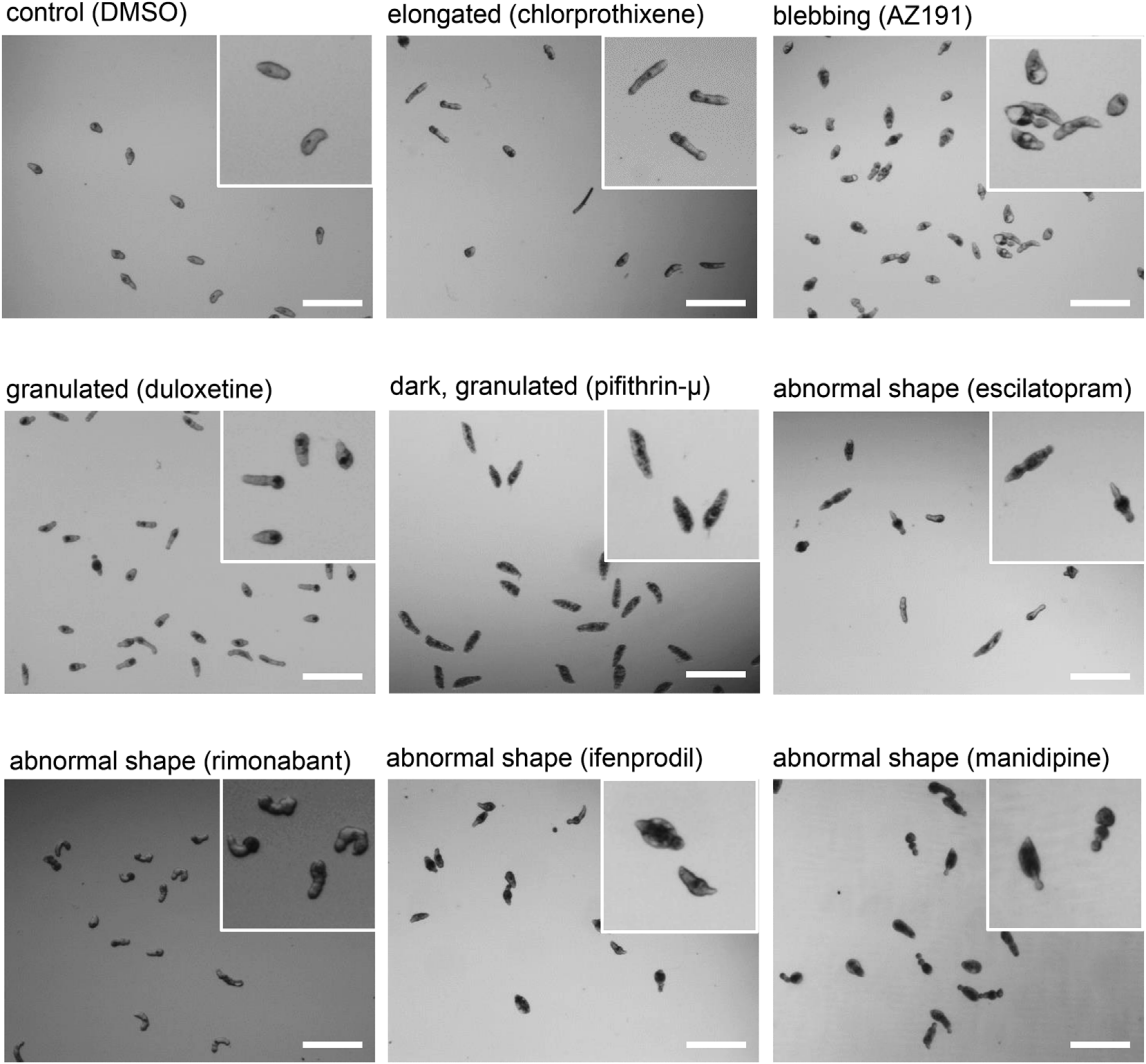
Compound-related morphological impact on schistosomulae. Three-day old shistosomulae were exposed for 24 h to 10 μM of each selected compound. Images were obtained five days post drug exposure. The images represent the main observable phenotypes in comparison to control animals treated with vehicle only (DMSO). Magnified caption is represented in the upper right corner of each image. Bars indicate 300 μm.

Comparison to control animals exposed only to DMSO, which exhibited clear ovoidal shape, revealed observable phenotypes with a majority (47 compounds) producing extensive internal dark granulation four days post exposure (Fig. 2, Sup. Table 2). Other drugs (14%) induced body elongation in schistosomulae, while the joint occurrence of a dark body and granulations was a common feature of dead parasites (Fig. 2). Notably, the phenotypic aspects of compound-treated schistosomulae did not seem related to pathway class as evidenced by the neuronal signaling group of molecules, which induced a variety of phenotypes, including enlarged, dark, blebbing, elongated and/or granulated parasites (Fig. 2, Suppl. Table 2).

These primary observations allowed us to select a set of hits associated with markedly reduced viability and/or induced hypo/hyper-motility phenotypes, leading us to conclude that changes in these two criteria are suitable end-points for a larger-scale assay on schistosomulae.

### 3.2 A motility assay on 24 h-treated schistosomulae highlighted a variety of movement impairment patterns and identified significant hits

Our selection of hits based on phenotypic observation represents a first step toward the establishment of a more quantitative ranking of motility profiles. We applied a motility calculation method previously developed in our laboratory for this purpose (Patocka et al., 2014). To attribute motility values to each drug, videos of schistosomulae and adult worms were captured after exposing them for 24 h to compounds (1 and 10 μM), followed by calculation of motility values that were normalized to control conditions (animals exposed to DMSO) (Sup. Table 3).

**Fig. 3:**
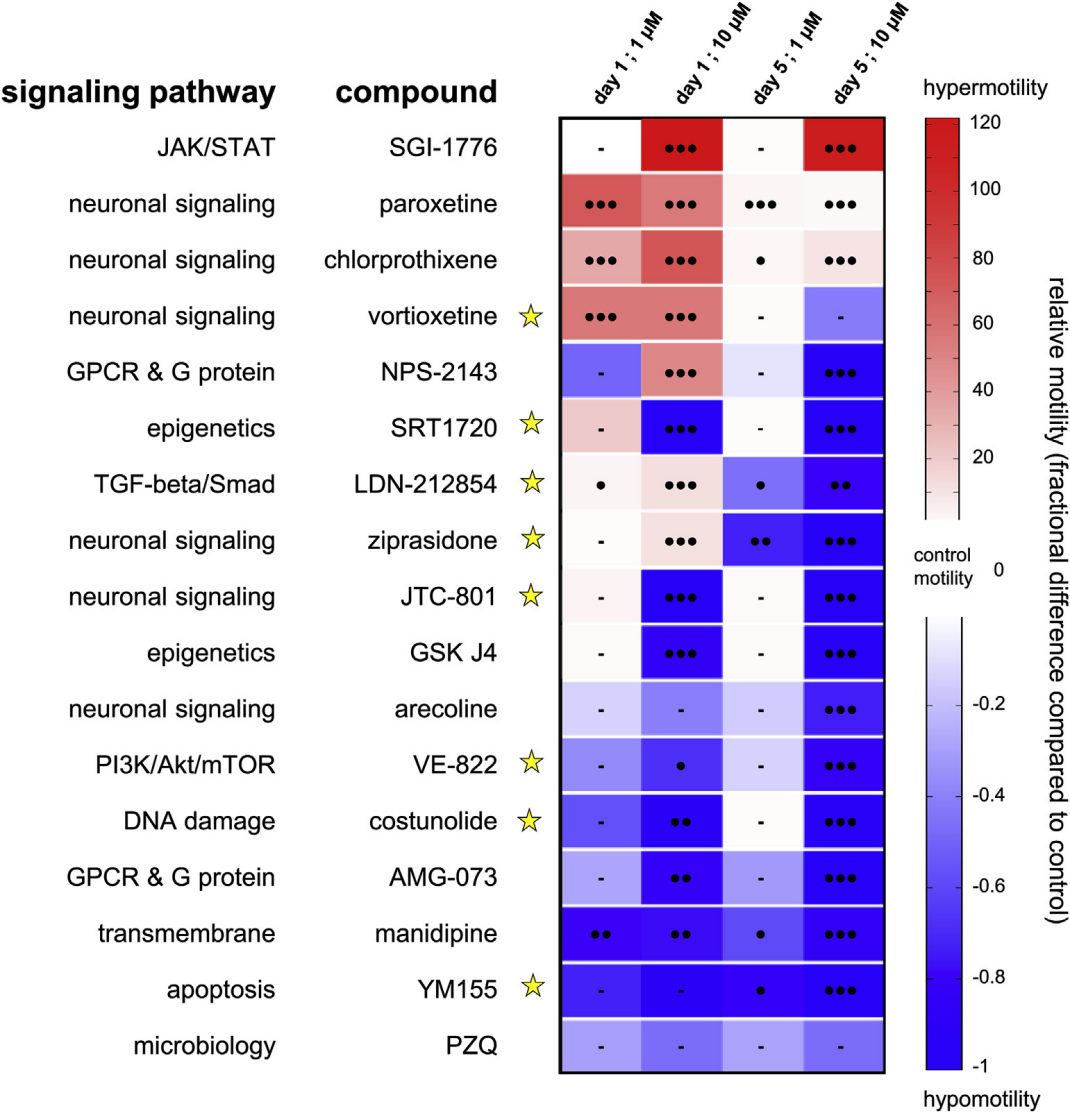
**Motility assay on schistosomulae treated with secondary screened compounds**. Heat map of all secondary screened compounds. Three-day old schistosomulae were treated with 1 and 10 μM of each compound and motility was calculated relatively to control animals (DMSO-treated). Blue and red colors correspond to hypo- and hypermotility respectively. Viability <50% after five days is represented as a yellow star. Black circles indicate p value < 0.0005 (3), <0.005 (2), <0.05 (1). Bars represent no significant change in motility rate compared to control.(For interpretation of the references to color in this figure legend, the reader is referred to the Web version of this article.)

Based on this approach, a variety of motility patterns was recorded for every molecule (Sup. Fig. 1); compounds exhibiting the most significant effects are presented in Fig. 3. For the 70 selected compounds, 51.4% induced significant hypermotility behavior at 10 μM after 24 h incubation, whereas 19% of them caused hypomotility (Table 1). Long term effects were also determined by washing out the drug from the culture medium followed by further incubation of parasites for up to five days in identical but drug-free conditions. In this screening set, hypomotility was more commonly observed than hypermotility (51.4 and 18.6% of compounds, respectively). Interestingly, 18.6% of the selected hits initially downregulating motility after 24h also induced a hypomotile behavior over time (Table 1).

**Table 1:** **Distribution of compounds per motility phenotype in *S. mansoni* schistosomulae adult stages.** Three-day-old schistosomulae were incubated with 10 μM compound for 24 h and motility recorded one and five days after compound application. Adult male and female parasites were exposed to 10 μM of each compound for 24 h and motility was recorded after compound incubation and seven days later. Numbers in brackets represent percentage of total selected compounds. * Total compounds for both sexes and reading timepoints. **N.S. indicates no significant change in motility compared to control animals.

	**Schistosomulae**			**Adults**						
	Day 1	Day 5	Day 1 & 5	Male Day 1	Female Day 1	Both Day 1	Male Day 7	Female Day 7	Both Day 7	Total Adults*
**Hypermotility**	36 (51.4%)	13 (18.6%)	11 (15.7%)	17 (23.9%)	18 (25.4%)	10 (14.1%)	3 (4.8%)	3 (4.8%)	0 (0%)	1 (1.4%)
**Hypomotility**	15 (21.4%)	36 (51.4%)	11 (15.7%)	8 (11.3%)	9 (12.7%)	4 (5.6%)	29 (46%)	37 (58.7%)	20 (31.7%)	2 (2.8%)
N.S.**	19 (27.1%)	21 (30%)	5 (7.1%)	46 (64.8%)	44 (62%)	34 (47.9%)	31 (49.2%)	23 (36.5%)	11 (17.5%)	9 (12.7%)

In schistosomulae, high levels of hypermotility were recorded for a small group of neuromodulatory molecules, including the GPCR antagonists vortioxetine and chlorprothixene as well as the antidepressant and serotonin re-uptake inhibitor paroxetine (Fig. 3). Interestingly, the last two were previously noted to have excitatory effects on schistosome motility at 10 μM (Neves et al., 2016; El-Sakkary et al., 2018). Although strongly activating contraction/relaxation cycles of the animals at both concentrations, this effect was lost four days after drug washout. Vortioxetine was the only schistosomulicidal molecule among the group causing strong hypermotility after 24 h that persisted five days after drug washout (Fig. 3). Only the serine/threonine kinase inhibitor SGI-1776 stimulated motility >100-fold throughout the course of the experiment (Fig. 3). In contrast, exposing schistosomulae to the Ca^2+^ channel blocker manidipine induced ≥59% hypomotility at all tested concentrations until five days after drug washout. In addition, a larger number of compounds strongly dampened motility at 10 μM, with some being lethal (*e.g*., the opiate receptor antagonist JTC-801 and the activator of NAD-dependent deacetylase sirtuin-1, SRT1720) (Fig. 3). Among the compounds that induced a shift from a hypermotility to hypomotility phenotype over time, the serotonin receptor antagonist ziprasidone induced 11 ± 2-fold hypermotility after 24 h incubation, whereas all parasites became paralyzed five days after compound washout (Fig. 3). Finally, the Ca^2+^-sensing receptor (CaSR) antagonist NPS-2143 stimulated movement up to 50-fold during the first 24 h followed by long-term, complete paralysis with no effect on viability of schistosomulae after 5 days (Fig. 3).

This confirmatory screen revealed a range of effects on schistosomulae motility by differentiating long from short-term effects associated with time-dependent hyper- and hypomotility phenotypes.

### 3.3 Adult stage motility assay identified long-term paralyzing compounds principally classified in the ubiquitin and transmembrane transporter-related pathways

**Fig. 4:**
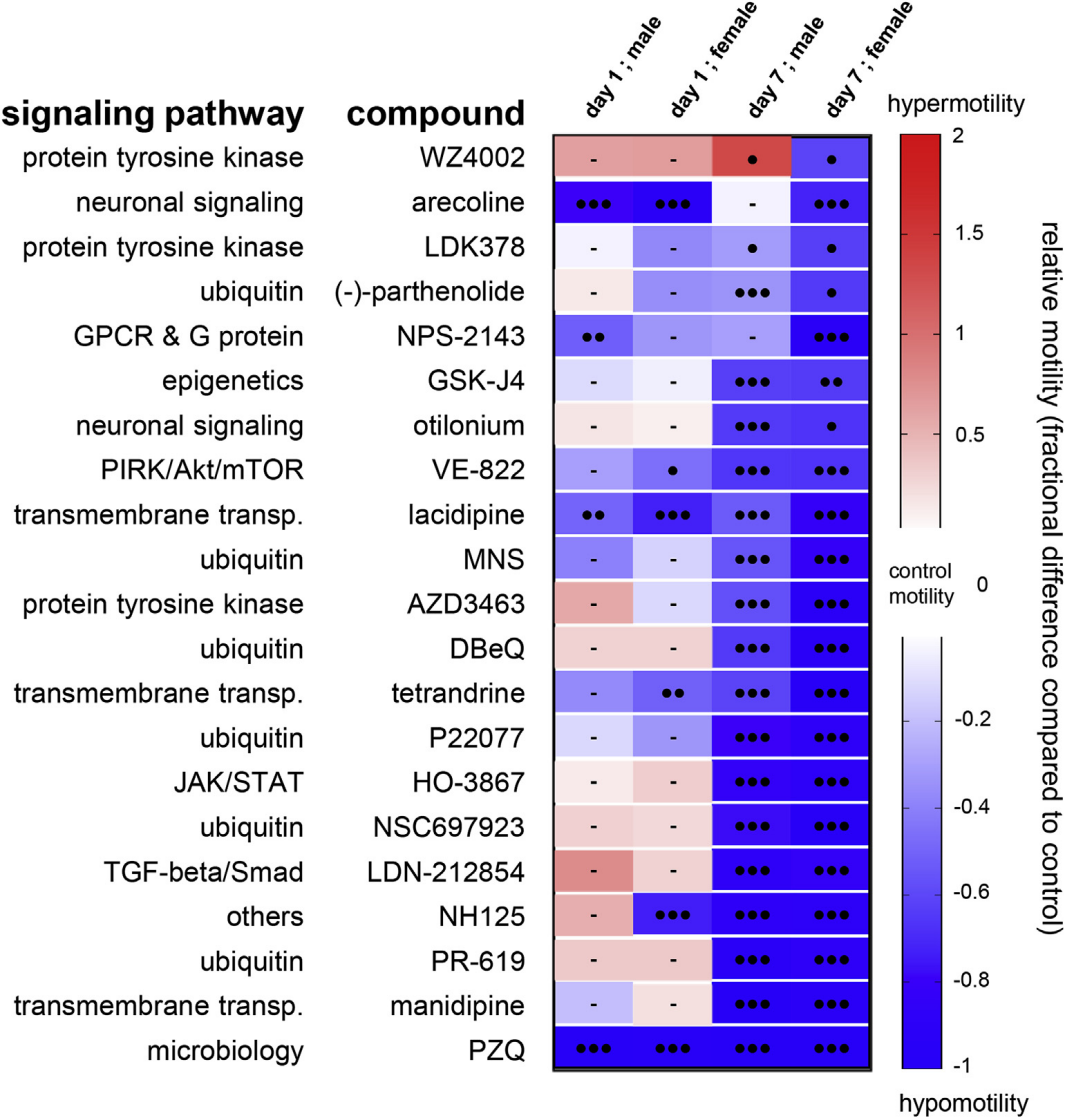
**Motility assay on adult parasites treated with secondary screened compounds.** Heat map of all secondary screened compounds. Motility rate was calculated relatively to control animals (DMSO-treated). Blue and red colors correspond to hypo- and hypermotility respectively. Black circles indicate p value < 0.0005 (3), <0.005 (2), <0.05 (1). Bars represent no significant change in motility rate compared to control. (For interpretation of the references to color in this figure legend, the reader is referred to the Web version of this article.)

Excepting eggs, all life stages of *S. mansoni* are motile, with variable degrees of motility and behaviours obeserved from schistosomulae to adults. To determine the effect of the selected compounds on adult worms, an approach similar to that for schistosomuale was applied with identical drug concentrations on worms perfused from mice three weeks after infection. After 24 h drug exposure, the motility of treated male and female worms was moderately affected compared to control parasites, with 47.9% of tested molecules causing no significant change (Table 1, Sup. Fig. 2). However, after washing the compound out and maintaining parasites in culture up to seven addtional days, modulation of motility rate was detected (Sup. Fig. 2). Whereas hypermotility never exceeded two-fold the control value for three hits, 46% and 58.7% of the compounds induced hypomotility at 10 μM for adult males and females, respectively, including 20 molecules (31.7%) that affected both sexes (Table 1). Interestingly, among the compounds affecting parasite motility (Fig. 4), only the EGFR inhibitor WZ4002 caused hypermotility in male worms and hypomotility in females.

For the 20 compounds slowing movement in both sexes (Table 1), the four ubiquitin-related drugs PR619, P22077, DBeQ and parthenolide were active on schistosomulae after five days incubation and severely paralyzed adults, with hypomotility ranging from 94.5% for PR619-treated male and female parasites to 49.5% in parthenolide-treated animals (Fig. 4). Similarly, the Ca^2+^ channel blockers manidipine and tetrandrine strongly impaired movement in both life-cycle stages in a long-term manner. Significant hypomotility of adult schistosomes was also recorded with 10 compounds previously identified as larvicides, including DBeQ, P22077 and the tyrosine kinase inhibitors AZD3463 and LDK378. The other similarly acting compounds LDN-212854, HO-3867, NSC697923, NH125, GSK-J4 and VE-822 appear to be related to distinct signalling pathways (Fig. 3, Fig. 4).

Other molecules with no chemical or pharmacological commonalities exclusively impaired adult motility, with negligible effects on schistosomulae. For example, the tyrosine kinase inhibitor MNS (3,4-methylenedioxy-β-nitrostyrene), the Ca^2+^ channel blocker lacidipine and the antimuscarinic otilonium did not significantly affect larval movement, whereas strong paralysis was observed in adults (53–83% motility inhibition compared to control) (Fig. 4).

This secondary screen highlights a set of compounds that affect adult motor behavior, with some molecules targeting both schistosomulae and adult worms with long-term effects.

To the extent that the effects of compounds observed *in vitro* mimic the *in vivo* situation under comparable exposures, selecting hits that act quickly *in vitro* may enhances the translatability of the activity into the mouse model. Thus, activity associated with reduced exposure times refined the activity patterns and enabled further selection of schistosomulicidal molecules.

**Fig. 5:**
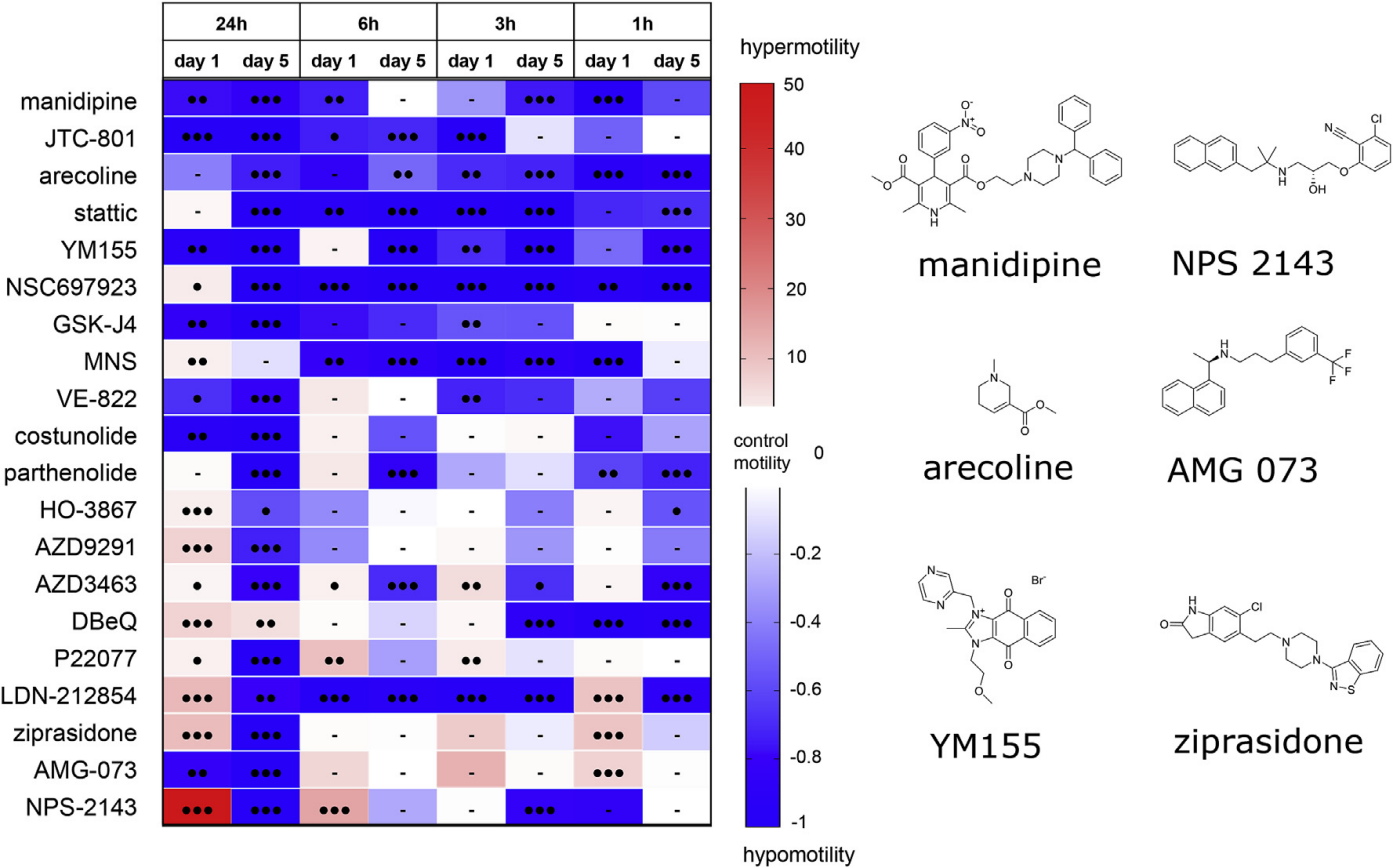
**Motility assay on schistosomulae treated with selected hits over decreasing incubation periods.** Heat map of compounds significantly impairing parasite motility (larval and adult stages). Motility assays were performed on three-day old schistosomulae and consisted of two motility recording time points (day 1 and day 5) following a 1–6 h compound incubation time. Motility rate was calculated relatively to control animals (DMSO-treated). Blue and red colors correspond to hypo- and hypermotility, respectively. Black circles indicate p value < 0.0005 (3), <0.005 (2), <0.05 (1). N.S indicates no significant change in motility rate compared to control. Shown on the right are chemical structures of key compounds showing hypo- and hypermotility patterns. (For interpretation of the references to color in this figure legend, the reader is referred to the Web version of this article.)

Fourteen molecules that significantly impaired motility of both life-cycle stages were tested in an extended motility assay on three-day old schistosomulae. A similar protocol was applied, but included stopping compound incubation after 6 h, 3 h and 1 h while keeping 24 h as a control condition. Motility recording revealed a set of fast-acting molecules, including manidipine, YM155 and LDN212854, which caused significant and sustained hypomotility (Fig. 5). The cholinergic compound arecoline induced a similar effect, matching previous obervations made at 100 μM (MacDonald et al., 2014). Interestingly, these molecules had long-term effects on schistosomulae for all tested incubation times. Other compounds, including VE822, costunolide, ziprasidone and AMG073, did not exhibit a significant change before the 24 hr incubation cut-off despite a strong motility effect observed at this time point (Fig. 3). Overall, these data support the conclusion that incubation time is a key parameter to predict hit compound efficiency *in vitro*. More importantly, this complementary assay highlights four additional compounds that match the requirement for fast and dramatic effects on parasite motility.

### 3.4 Chemical analogs of the anthelmintic arecoline and NPS-2143 inhibit *S. mansoni* motility

**Fig. 6:**
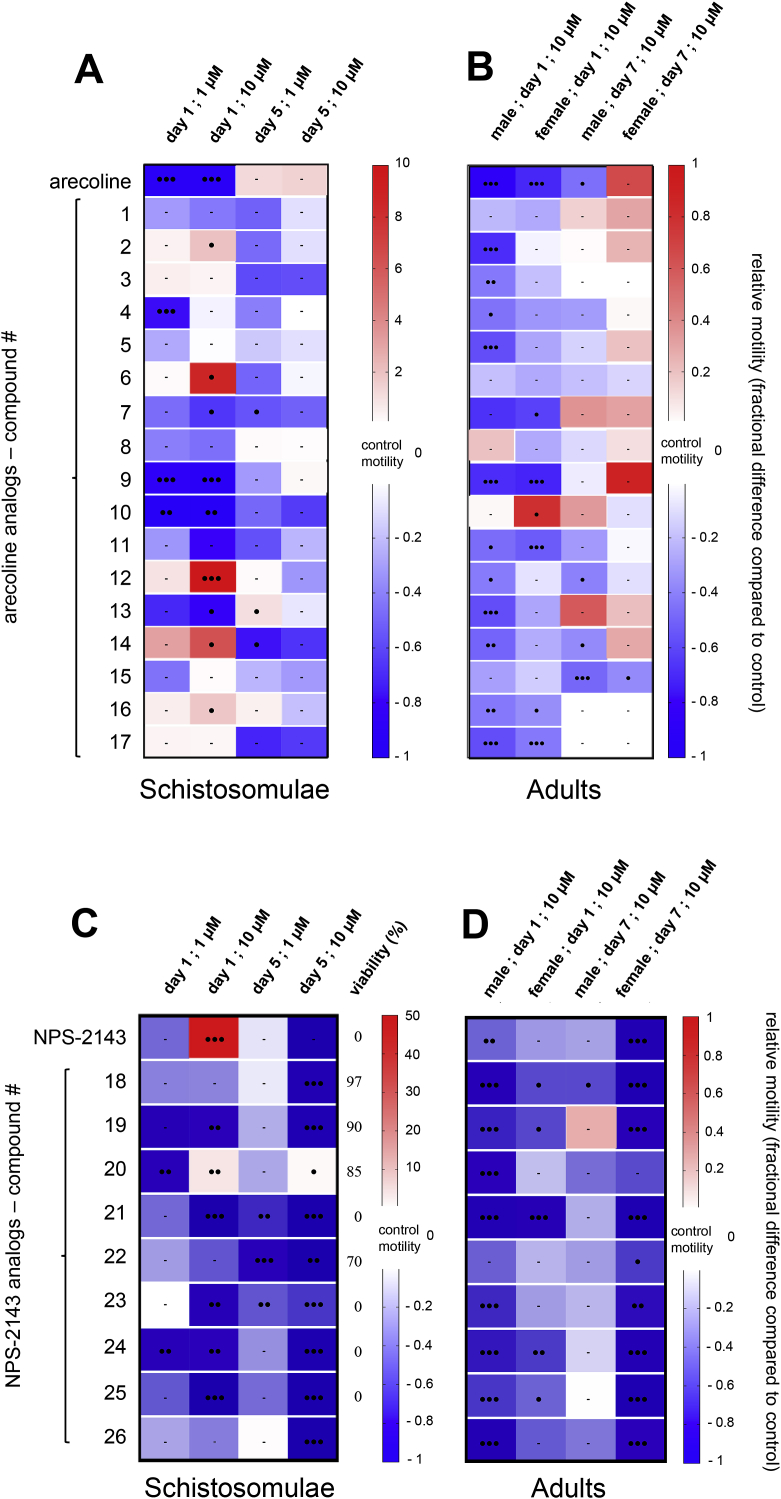
**Motility assay on *S. mansoni* schistosomulae and adults treated with arecoline, NPS-2143 and respective chemical derivatives.** Heat maps of selected arecoline (A, B) and NPS-2143 (C, D) chemical analogs tested at 1 and 10 μM on three-day old schistosomulae and at 10 μM on adult worms. Similar experimental conditions used for the secondary screen of Selleckchem® compounds were applied (Fig. 3, Fig. 5). Motility rate was calculated relative to control animals (DMSO-treated). Blue and red colors correspond to hypo- and hypermotility respectively. Black circles indicate p value < 0.0005 (3), <0.005 (2), <0.05 (1). Bars represent no significant change in motility rate compared to control. When indicated, viability percentage was determined on larvae, five days post compound exposure. (For interpretation of the references to color in this figure legend, the reader is referred to the Web version of this article.)

Neuronal signaling molecules represent a large fraction of compounds in the customized Selleckchem® compound library that were active in the schistosomula motility assay. Among them, arecoline is an old intestinal purgative used as a canine anthelmintic and diagnostic tool for echinococcosis (Gemmell, 1973), which induces flaccid paralysis in schistosomes with negligible schistosomulicid effects (Mellin et al., 1983; MacDonald et al., 2014). To explore this anti-schistosomal activity, 17 arecoline derivatives (Sup. Table 4) were tested on schistosomulae and adult parasites in motility assays. As shown in Fig. 6A and B, the prototype arecoline induced short term, pronounced paralysis in both life stages. This effect rapidly attenuated, with normal movement regained within five-seven days. The majority of the 17 analogs did not impair motility or viability of schistosomulae. However, derivatives **10** and **9** strongly paralyzed animals at 1 μM (respective motility decreases of 87 ± 44% and 100%) (Fig. 6A). In contrast, a number of analogs induced a hypomotile effect on adult parasites. The parent molecule caused nearly complete paralysis in male and female adult worms (Fig. 6B), and four analogs (compound 9, 11, 16 and 17) also led to a hypomotility phenotype after 24 h. Only analog **15** induced a unique long-term effect with motor activity reduced by 49.1 ± 0.1 and 37.1 ± 0.1% in male and female worms, respectively (Fig. 6B). Interestingly, among all analogs, **9** caused a significant hypomotile effect on both life-cycle stages, which reversibly evolved to a normal motility rate at the time of the second motility recording seven days after incubation.

Considering NPS-2143 and its intriguing effect on schistosomulae motility, 302 chemical analogs were examined. An initial screen identified 20 analogs (Sup. Table 4) that induced visible dark granulations and/or paralysis at 10 μM on schistosomulae; 14 of them induced a complete schistosomulicidal effect at five days (Sup. Fig. 3, Sup. Table 3). As the number of adult schistosomes became limited at this time, compounds **18** to **26** were tested on both life-cycle stages for comparative analysis at 1 and 10 μM (Fig. 6C and D). Five analogs induced animal death five days after compound washout. Reducing the concentration to 1 μM led to a strong and long-term hypomotile phenotype for analogs **20** and **24** (Fig. 6C). Other compounds, such as **19** and **22**, did not impact larval viability but considerably attenuated the motility rate to near complete paralysis in an irreversible manner (Fig. 6C). In comparison to the parent molecule, which induced a hyperexcitability state after 24 h incubation, compound **20** was the sole analog sharing similar properties but with a hypermotility state remaining 4.1 ± 0.9-fold higher than control animals (Fig. 6C). Testing the same set of compounds in adult worms led to a singular short term but strong decrease in male motility after 24 h incubation at 10 μM. However, female worms were significantly impacted in a long-term manner, with worm paralysis recorded at seven days (Fig. 6D).

This motility assay highlights the relevance of screening analogs of known paralyzing agents, as stronger effects can be obtained *in vitro* that support interest in screening potential molecular targets. Taken altogether, the compilation of all secondary screened compounds and derivatives led to establishing a candidate pool of compounds (Sup. Table 5) prioritized for *in vivo* testing and subsequent analysis on parasite neuromuscular system drug targets.

### 3.5 Target compound profiling of key *S. mansoni* G-protein coupled receptors

With the ultimate goal of testing candidate molecules *in vivo*, we first investigated their activity on a set of key neuromodulatory *S. mansoni* GPCRs previously identified by the group of late Dr. Paula Ribeiro (Taman and Ribeiro, 2009; El-Shehabi and Ribeiro, 2010; Patocka et al., 2014). In addition, a set of 11 molecules with demonstrated activity *in vitro* and/or *in vivo* against *S. mansoni*, including chlorpromazine and arecoline, which our group previously identified as altering motility (MacDonald et al., 2014; El-Sakkary et al., 2018), was included in the target-compound profiling process (Table 2). We also included praziquantel, stemming originally from an antipsychotic research programme, and other drugs with human neuropharmacology; we hypothetized that these compounds could represent putative *S. mansoni* GPCR modulators, allowing us to expand the relevant target space in schistosomes.

**Table 2:** Compounds with reported activity against schistosomes *in vitro* and/or in vivo

Compound	Name	Class	Reference(s)
	(*R*)-PZQ	anthelmintic^a^	(Olliaro et al., 2014; Kovac et al., 2017; Vale et al., 2017)
	(*S*)-PZQ	anthelmintic^a^	
	Arecoline	anthelmintic^b^	(Mellin et al., 1983; Day et al., 1996; MacDonald et al., 2014; El-Sakkary et al., 2018)
	Chlorpromazine	antipsychotic	(El-Sakkary et al., 2018; Weeks et al., 2018)
	Trifluoperazine	antidepressant	Panic et al. (2015b)
	Paroxetine	antidepressant	(Neves et al., 2016; Weeks et al., 2018)
	Mefloquine	antiplasmodial	Van Nassauw et al. (2008)
	Flunarizine	antihypertensive	Panic et al. (2015b)

a Discovered as a racemate from a discontinued antipsychotic drug discovery programme.

b An old intestinal purgative used as a canine anthelmintic and diagnostic tool for echinococcosis.

**Fig. 7:**
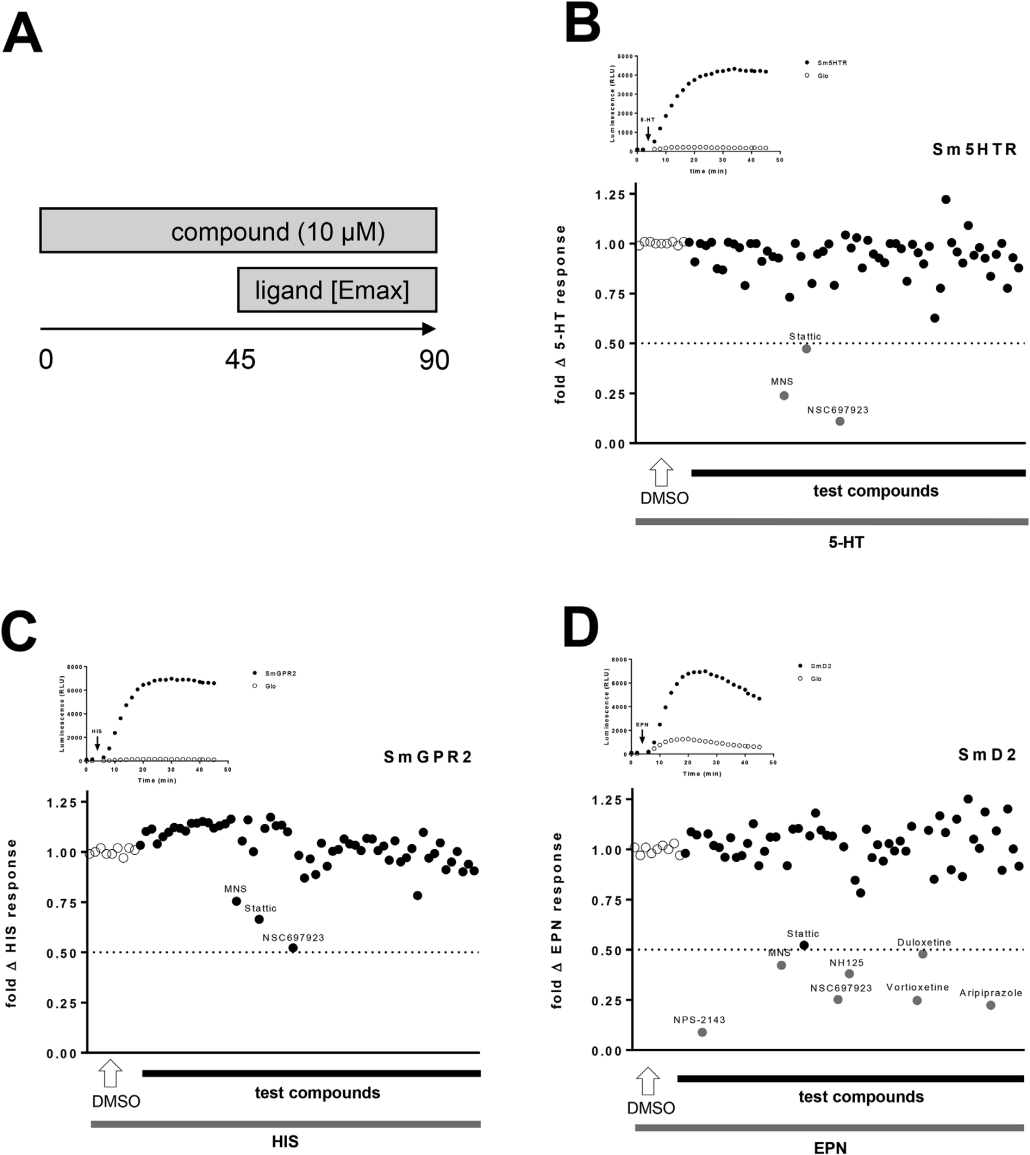
**Compound/target profiling on *S. mansoni* Sm5HTR, SmGPR2 and SmD2 receptors.** Stable HEK293 cell lines expressing *S. mansoni* GPCRs were generated and maintained in G418 selection conditions. (A) Schematic of the cAMP luminescent assay integrating potential agonist and antagonist properties of all tested compounds. (B–D) Scatter plots representing fold change luminescence response of all test compounds on HEK293 cells expressing *S. mansoni* GPCRs in comparison to DMSO-treated (control) cells. A 50% inhibition threshold was set to define ‘‘hits'’ as proposed by Chan and colleagues (Chan et al., 2016). Inset kinetic luminescence curves were performed at 50 μM agonist concentration.

Among these, the serotonin sensitive Sm5HTR (Smp_126730), the histaminergic SmGPR2 (Smp_043340) and the dopaminergic SmD2 (Smp_127310) receptors represented the best characterized and most suitable candidate GPCRs for target/compound profiling. Stable expression in HEK293 cells was performed to ensure better reproducibility of results and the presence of the 3 receptors was validated by immunofluorescence (Sup. Fig. 4A). To measure receptor activity, the GloSensor technology, which provides live cell luminescent cyclic AMP accumulation reading, was adopted, as previously shown for Sm5HTR (Chan et al., 2016). Following the recording protocol detailed in Fig. 7A, both Sm5HTR and SmGPR2 induced recordable luminescent signals under exposure to 5-HT (EC_50_ = 1.46 ± 0.13 μM) and histamine (EC_50_ = 12.53 ± 1.5 μM), respectively (Fig. 7B and C). The robustness of the assay was calculated based on the minimal response of naïve cells (GloSensor only) as detailed (Chan et al., 2016) with Z’ factors of 0.77 (Sm5HTR) and 0.72 (SmGPR2), respectively. None of the selected compounds increased cell luminescence over background, as opposed to >4000 RLU signals obtained with 5-HT and histamine (Fig. 7B and C). However, compound incubation followed by 50 μM 5-HT identified three molecules that inhibited Sm5HTR 5-HT-evoked signals by ≥ 50%: the human STAT3 inhibitor Stattic, the tyrosine kinase inhibitor 3,4-methylenedioxy-β-nitrostyrene (MNS) and the E2 ubiquitin-conjugating enzyme inhibitor NSC697923 (Fig. 7B). The same compounds inhibited luciferin enzyme activity in naïve cells, as a significant decrease in luminescence evoked by 20 μM forskolin was recorded (Sup. Fig. 4B). Moreover, NSC697923, in contrast to any other compound used in these assays, was also toxic, reducing the ATP released by cells by 48.1 ± 5.7% (Sup. Fig. 4C).

**Fig. 8:**
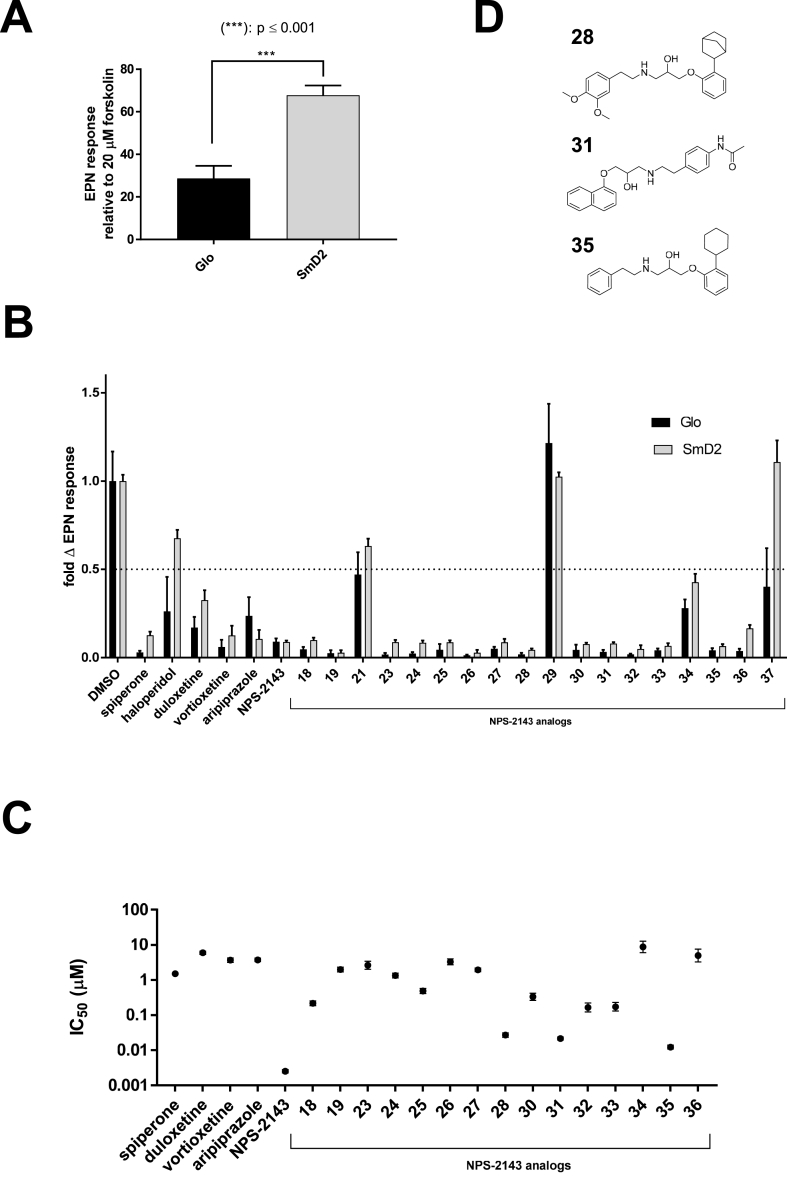
**Similarity of EPN inhibition responses between SmD2 and naïve (Glo) cells.** (A) SmD2 expressing cells and control Glo cells were exposed to 50 μM epinine (EPN) and luminescence responses were compared to 20 μM forskolin exposure. *** indicates p < 0.001 according to a Student's t-test. (B) Previously identified hits and *in vitro* screened NPS-2143 analogs were exposed on SmD2 and Glo cell lines at a 10 μM concentration and fold change EPN responses were calculated compared to DMSO-treated cells. (C) 10^−10^ to 5.10^−5^ M concentration-responses analysis was performed with all hits on SmD2 expressing cells. Error bars indicate SD. (E) Chemical structure of NPS-2143 analogs exhibiting the most potent inhibition of SmD2 EPN-evoked luminescent signals.

The dopaminergic GPCR SmD2 was less adaptable for compound screening, as HEK293 cells exhibited an intrinsic response to dopamine (DA) and to a higher extent to epinine (EPN) through the putative activation of endogenous GPCRs (Fig. 8A, Sup. Fig. 4D). The responses observed with 50 μM EPN were, however, significantly increased (p < 0.001) in cells expressing *S. mansoni* receptors, but the Z’ factor remained as low as 0.19 and the EPN EC_50_ was 2.52 ± 1.12 μM (Sup. Fig. 4E). Considering each of the candidate compounds, no stimulatory activity was recorded, but a ≥50% inhibition of EPN-evoked luminescence was obtained with the antidepressant duloxetine and vortioxetine as well as with the antipsychotic aripiprazole on both naïve and SmD2 cells. This effect was concentration-dependant and the IC_50s_ was in the micromolar range (Fig. 8C, Sup. Table 6).

Interestingly, the calcium receptor inhibitor NPS-2143 caused a dramatic drop in EPN-evoked luminescence (91.1% at 10 μM; Fig. 7D). A concentration-response assay revealed 100-fold higher sensitivity compared to the previous hits, with IC_50_ of 2.53 ± 0.28 nM (Fig. 8C, Sup. Table 6). Subsequent testing of NPS-2143 analogs that impaired parasite motility revealed 15 analogs that inhibited (>50%) EPN-evoked luminescence in a similar manner in all cell lines (Fig. 8B) and eight of these analogs exhibited IC_50s_ within the nM range; compounds **28**, **31** and **35** were the most potent (Fig. 8C, Sup. Table 6). Noteworthy, compound **19** seemed to directly inhibit forskolin-induced signals through the activation of endogenous receptors that decrease cAMP levels, such as Gi-GPCRs, or direct inhibition of the Glo-Sensor activity (Sup. Fig. 4B).

Taken as a whole, receptor profiling failed to identify *S. mansoni*-specific compound/target interactions, but revealed broad-spectrum activity of NPS-2143 and related analogs on dopamine/EPN-sensitive GPCRs.

## 4 Discussion

In the present study, a compound library of bioactive molecules with known mammalian targets was tested for activity against schistosomes in culture. This strategy was hypothesized to facilitate subsequent research on the mode of action of potential hits through the identification of their molecular targets. Moreover, this work demonstrates the importance of using two developmental stages of the parasite simultaneously to refine the prioritization of hits according to the desired therapeutic objectives of activity against both larval and adult-stage parasites in the human host.

The set of 708 compounds was assembled based on past research performed *in vitro*, primarily focused on the impairment of neuromuscular activity of *S. mansoni* (Ribeiro and Geary, 2009; MacDonald et al., 2014; El-Sakkary et al., 2018), and other pathways that impair parasite development and behavior. These compounds have been extensively studied in the context of their potential human therapeutic use, and this information represented a treasure trove for further exploitation of potential hits for drug discovery, in contrast to an unbiased chemical library. Drug repurposing/repositioning represents an interesting alternative (Ramamoorthi et al., 2015) to costly *de novo* drug discovery and has already proven its relevance for helminth drug discovery, as exemplified by praziquantel, a drug that stems from a library of compounds originally developed for a potential antipsychotic indication.

The primary screen of a large subset of molecules on schistosomulae as indicators of activity against juvenile stages echoes numerous studies that used thousands of newly transformed schistosomulae to reach HTS (Abdulla et al., 2009; Asarnow et al., 2015; Tekwu et al., 2016; Lombardo et al., 2019). Indeed, this approach allows compound triaging, while enabling multiple experimental conditions and reducing the use of mammals in experiments (Maccesi et al., 2019).

The measurement of motility of treated *versus* untreated schistosomulae highlighted an extreme range of movement speeds, which can lead to better characterization of compound activity. However, the movement rate of adult parasites is reduced in amplitude, necessitating careful attention to the maintenance conditions to ensure accuracy and reproducibility. A wide range of morphological changes was observed in schistosomulae, which allowed the screening process to be guided in a standardized way. This strategy may have omitted compounds that act exclusively on adult worms, but is consistent with the aforementioned therapeutic goals.

The primary and secondary *in vitro* screening performed on schistosomulae led to the refinement of the criteria used for identification of molecules that affect parasite viability and/or motility. Previously reported methods (Ingram-Sieber et al., 2014; Panic et al., 2015b) would have identified only 18 molecules that diminished viability by >25%. In contrast, our procedur identified many more interesting hits. Among them, two compound clusters related to human ubiquitin and neuro-signalling pathways may reveal key targeting strategies applicable to these parasites. Indeed, whereas a majority of anthelmintics modulate helminth neurotransmission (Holden-Dye and Walker, 2007), targeting the ubiquitin proteasome represents a new stragegy against protozoan parasitic diseases such as malaria and trypanosomiasis (Bibo-Verdugo et al., 2017; Gupta et al., 2018; Pereira et al., 2018). The possibility of extrapolating this strategy to schistosomes is gaining attention as illustrated by the proteasome inhibitors bortezomib and carfilzomib, which reduce worm motility (>85%) and proteasome activity (>75%) *in vitro* (Bibo-Verdugo et al., 2019). Interestingly, in mice treated with the proteasome inhibitor MG-132, 65% fewer parasites migrated to the lung compared to control animals (Guerra-Sa et al., 2005). In addition, RNAi-mediated knockdown of a deubiquitinase decreased schistosome viability by 78% (Nabhan et al., 2007). The motility of schistosomes treated with other proteasome inhibitors such as bortezomib and carfilzomib can also be severely impacted (>90% reduction; (Marcellino et al., 2012), which is supported by the dramatic reduction in motility and/or viability we observed with most of the selected ubiquitin-related compounds. The fact that MNS and NSC697923 unexpectedly inhibited several *S. mansoni* GPCRs indicates broad spectrum functions that may involve multiple targets. Indeed, the example of MG-132, which regulates the expression of up to 1,919 genes in adult worms (Morais et al., 2017), supports this hypothesis.

Interrogating both schistosomulae and adult worms in identical conditions allowed us to highlight life cycle stage-specific drug action. The hypermotility observed with all selected antipsychotic and antidepressant compounds in schistosomulae is an example. Among these non-lethal molecules, paroxetine induced a rapid and transient effect on the motility of schistosomulae. Interestingly, these data partially corroborate previous findings demonstrating a transient hyperactivity state of paroxetine-treated adult schistosomes with EC_50s_ varying from 2.7 to 11.9 μM in male and female worms, respectively (Neves et al., 2016). While signs of hypermotility were also observed in our assay, the difference compared to control animals was not statistically significant and may be explained by the longer incubation time (72 h) used in the study. More importantly, while the dramatic hyperexcitability of schistosomulae observed at 1 and 10 μM was unreported by Neves and colleagues, such a phenotype may correspond to a serotonin excess induced by paroxetine, a known serotonin reuptake inhibitor (Bourin et al., 2001). Similarly, our assay also highlighted chlorpromazine as causing >10 fold hypermotility compared to control schistosomulae at 10 μM. Most recent studies corroborate these data (Abdulla et al., 2009; Weeks et al., 2018), as shown by El-Sakkary and colleagues, who demonstrated that 20 min exposure to this concentration induced a peak exceeding 50-fold the motility of control schistosomulae but that was rapidly followed by paralyzis at higher concentration (El-Sakkary et al., 2018). Paralysis may be the consequence of effects on multiple chlorpromazine-sensitive neuromodulatory receptors, but supports the rationale of screening molecules at lower concentrations to decipher multiple compound-related effects.

The same reasoning led us to take advantage of the cholinomimetic action of arecoline, which rapidly inhibits parasite movement in both life-cycle stages (Mellin et al., 1983; MacDonald et al., 2014). Indeed, at 100 μM, acetylcholine, arecoline and nicotine rapidly induce muscular relaxation of schistosomulae, notably through the activation of acetylcholine-gated chloride channels (ACCs) (MacDonald et al., 2014). Longer incubation times at 10 μM or lower concentrations revealed a sustained hypomotile effect that persisted after drug removal, which supports testing the interest of exploring long-term effects of neuromodulatory compounds on worm behavior. However, the fact that very few arecoline analogs showed effects similar to the prototype may reflect a narrow structure-activity relationship (SAR). While the molecular target of arecoline in schistosomes may involve ACCs (MacDonald et al., 2014), other muscular cholinergic ligand-gated ion-channels (Day et al., 1996) as well as ACh-sensitive GPCRs (MacDonald et al., 2015) may be involved and the specific receptor(s) need to be identified through a receptor deorphanization strategy (Weth et al., 2019). This approach may certainly enable future SAR studies and elucidate whether some analogs are more active than others. This complexity of targets merits consideration when observing behavioural phenotypes, as the overall inhibitory effect of arecoline may result from effects on multiple receptors. Indeed, like arecoline, ACh downregulates muscular activity (Day et al., 1996) and causes flaccid paralysis (Barker et al., 1966) but activates receptors with opposite effects on worm motility such as SmACCs (inhibitory) (MacDonald et al., 2014) and the cholinergic GPCR SmGAR (stimulatory) (MacDonald et al., 2015).

Whereas the secondary screening process identified a majority of compounds with unreported activity on schistosome motility, others effects were previously noted in the literature and hence support the robustness of our approach. Indeed, the histone demethylase inhibitor GSK-J4, shown here to dramatically inhibit larval and adult motility, was also reported with similar motility recordings and schistosomulae phenotype at 10 μM but over a longer and continuous incubation period (72h) (Whatley et al., 2019). These discrepancies of experimental processes (concentration, incubation time) are often observed throughout screening studies and can complicate conclusions on the effect of a particular active compound. In this study and despite a limited number of animals imposed by the size of our screening, we opted for two concentrations and two recording time points to enable comparative analysis with other published studies on identical or similar compound types. For example, the antihypertensive manidipine was recently validated as schistosomulicidal over 72 h incubation at 10 μM (Panic et al., 2015b). As our protocol limited compound incubation to 24 h, no larval mortality was observed, but the complete paralysis recorded on adults confirms the previous report of adulticidal activity after 24 h (Panic et al., 2015b). The plant-derived sesquiterpene lactone parthenolide caused severe tegumental damage and 100% mortality at 12.5–100 μM (de Almeida et al., 2016). Our data indicate long-term hypomotility at a lower concentration (10 μM) and provide new evidence of a total schistosomulicid action after 24 h incubation.

NPS-2143 and its analogs provided promising results for prolonged action on parasite motility and viability. In addition, the abundant analogs available were used to generate preliminary SAR data that could lead to addressing the identification of potential druggable targets in schistosomes. However, based on our data, it is difficult to hypothesize about the mode of action of NPS-2143 on schistosomes and whether the observed effects result from disruption/modulation of multiple targets and signaling pathways. Interestingly, a key feature of PZQ is the disruption of calcium balance in schistosomes through the modulation of several Ca^2+^-related protein targets (Salvador-Recatala and Greenberg, 2012). These notably include voltage-operated Ca^2+^ channels (Kohn et al., 2001), Ca^2+^ signaling kinases CamKII (You et al., 2013) and the recently identified Ca^2+^-permeable transient receptor potential (TRP) (Park et al., 2019), whose disturbance of Ca^2+^ balance ultimately induces paralysis (Cioli and Pica-Mattoccia, 2003; Vale et al., 2017; Thomas and Timson, 2020). Similarly, NPS-2143 may affect multiple receptors or bind to a principal target responsible for a broad range of effects. In humans, NPS-2143, antagonizes the CaSR, a class C GPCR that plays a central role in maintaining Ca^2+^ homeostasis (Kos et al., 2003; Ward and Riccardi, 2012) and has anticancer (Joeckel et al., 2014) and anti-inflammatory properties (Mine and Zhang, 2015). However, no CaSR-like ortholog has been identified in schistosomes despite an existing cluster of such receptors belonging to class C GPCRs in *C. elegans* (Nagarathnam et al., 2012). Interestingly, the interrogation of *S. mansoni* class A GPCRs and the intriguing inhibition of EPI-evoked signals by NPS-2143 and analogs may reflect a broader spectrum activity not yet reported. Testing these compounds *in vivo* could determine whether the schistosomicidal activity is equivalent to PZQ (assuming suitable pharmacokinetic properties), but such a test would also need to address potential side effects to position these compounds for drug development. NPS-2143 administered at 1 mg/kg significantly increases blood pressure in normotensive rats (Rybczynska et al., 2006) and may represents a concern for future application as anthelmintic. However, this issue could be overcomed by implementing lower doses, as NPS-2143 IC_50_ on human CaSR transiently expressed in HEK293 cells is as low as 43 nM (Nemeth et al., 2001), while its effect on *S. mansoni* EPI- responsive GPCRs was 10-fold less potent. While drug repurposing would bypasse some key drug development steps, NPS-2143 remains not FDA certified yet, which implies the implementation of complementary studies to elucidate its effect on parasitic GPCRs. Also, none of the *in vitro* active analogs has been tested in a laboratory animal model; whether any has significant activity on parasites with minimal side effects remains to be determined. *In silico* docking of these analogs on the human CaSR as well as on the human D2 receptor and its ortholog SmD2 would be a further prerequisite to address the questions highlighted in this study.

Some GPCRs and ligand-gated ion-channels play a central role in neurotransmission, as their stimulation or inhibition results in dramatic behavioral and motility changes in schistosomes (Hamdan et al., 2002a; Taman and Ribeiro, 2011; El-Shehabi et al., 2012; MacDonald et al., 2014, 2015; Patocka et al., 2014; El-Sakkary et al., 2018). We tested a limited number of receptors that are representative of three major neurotransmitters in the *S. mansoni* nervous system, 5-HT, histamine and dopamine. Other receptors identified by the group of Ribeiro and colleagues, such as the glutamatergic SmGluR (Smp_012620) (Taman and Ribeiro, 2011), the cholinergic Gi receptor SmGAR (Smp_145540) (MacDonald et al., 2015) and the histamine-sensitive SmGPR-1 (Smp_043260) (Hamdan et al., 2002a) were also expressed in HEK293 cells, but were not suitable for screening because of poor signal-to-background results (data not shown). The challenges of functionally expressing *S. mansoni* proteins in mammalian cells are well-known (Hamdan et al., 2002a, 2002b) and remain a major barrier for the optimization of cell-based functional screening assays. Despite the fact that no compound affected the function of Sm5HTR and SmGPR2, the relevance of considering these two receptors for large scale studies is supported by the activity of many antipsychotic, antidepressant and anxiolytic compounds that target neurotransmitter signalling systems. In this case, the interrogation of a dopaminergic GPCR was thought to echo the numerous compounds modulating Sm5HTR activity. Indeed, Chan and colleagues highlighted the effects of ergot alkaloids such as ergotamine as specific Sm5HTR agonists and motility disrupters (Chan et al., 2018; Marchant et al., 2018). Once used clinically for a wide range of indications, this group of compounds was shown to be unacceptably broad spectrum GPCR ligands (Dosa et al., 2013), but became of major interest regarding schistosomiasis treatment development (Ribeiro et al., 2012; Chan et al., 2015). Similarly, libraries of GPCR inhibitors also revealed the selective inhibitory effect of rotundine and atomoxetine, among others, on Sm5HTR, with poor activity on human Hs5HTR7 (Chan et al., 2016). As SmD2 responses were inhibited by vortioxetine and duloxetine, known human serotoninergic antagonists, broad *S. mansoni* target profiling was necessary to decipher these host/parasite pharmacological discrepancies. The challenging question of the presence of endogenous dopaminergic receptors in HEK293 cells (Atwood et al., 2011) must be addressed to guarantee the suitability of SmD2 and others for HTS.

The identification of hits with activity against schistosomes offers potential new starting points for drug discovery efforts. This study reports preliminary evaluation of compounds with *in vitro* schistosomulicidal effects, their impact on adult motility and their potential adverse effects on parasite development. More generally, further investigation of their mechanisms of action should include transcriptional analysis of treated parasites (You et al., 2013) in the case of molecules that are likely to impact multi-target networks. Alternatively, the use of gene-knockdown methods such as siRNA or CRISPR (Ittiprasert et al., 2019) on appropriate orthologous gene targets will, in combination with the aforementioned intiative, deepen our understanding of schistosome biology and help to open new drug discovery fronts against schistosomiasis.

## Contribution of authors

PR conceived and started this project in collaboration with TS, which was then continued by TBD, who analyzed parts of schistosome motility data and designed, performed and analyzed GPCR-related experiments. AG and AH maintained parasite culture, designed and performed parasite motility related experiments and contributed to the majority of motility data analysis. AG developed and maintained mammalian cell culture and GPCR expression systems. MR provided technical assistance as implementing motility recording methods. SK and IM provided project oversight and assistance in chemistry-related parts of the study. PR, TGG and TS provided project oversight and TBD wrote the manuscript. All authors, except PR, edited and proofed drafts of the manuscript.

## Declaration of competing interest

T.S is an employee of Ares Trading SA, an affiliate of Merck KGaA, Darmstadt, Germany. LSK and IM are employees of EMD Serono, a business of Merck KGaA, Darmstadt, Germany.
