# Supplementary material for: Identification of annotated bioactive molecules that impair motility of the blood fluke Schistosoma mansoni
Source: Int J Parasitol Drugs Drug Resist. 2020 Jun 1;13:73–88. doi: 10.1016/j.ijpddr.2020.05.002 (PMC7284125; doi:10.1016/j.ijpddr.2020.05.002)
Supplement: Multimedia component 1 [file mmc1.pptx]

## Slide 1
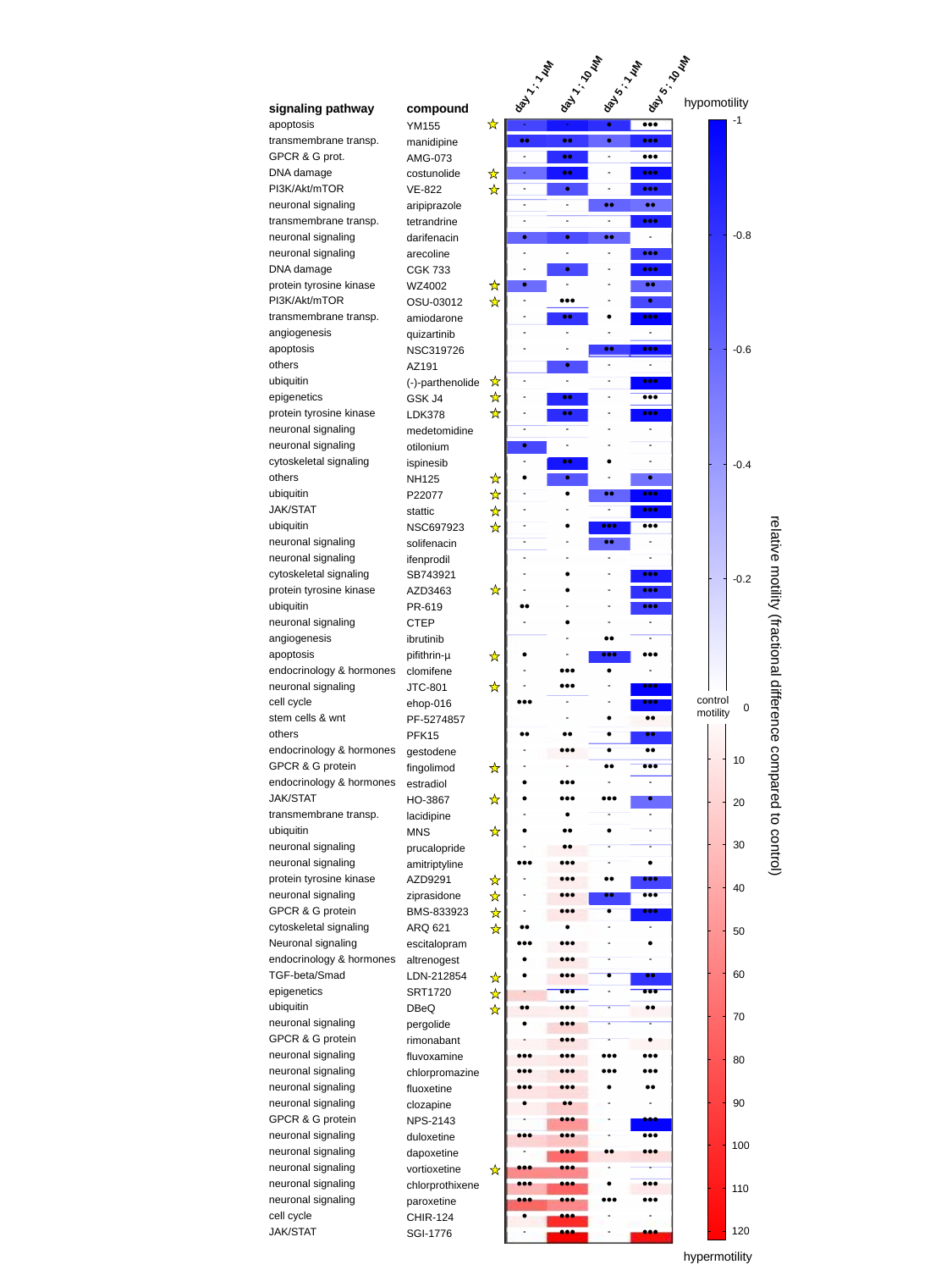

day 1 ; 1 µM
day 1 ; 10 µM
day 5 ; 10 µM
day 5 ; 1 µM
hypermotility
120
110
100
90
80
70
60
50
40
30
20
10
control motility
0
relative motility (fractional difference compared to control)
-0.2
-0.4
-0.6
-0.8
-1
hypomotility
| signaling pathway | compound |
| --- | --- |
| apoptosis | YM155 |
| transmembrane transp. | manidipine |
| GPCR & G prot. | AMG-073 |
| DNA damage | costunolide |
| PI3K/Akt/mTOR | VE-822 |
| neuronal signaling | aripiprazole |
| transmembrane transp. | tetrandrine |
| neuronal signaling | darifenacin |
| neuronal signaling | arecoline |
| DNA damage | CGK 733 |
| protein tyrosine kinase | WZ4002 |
| PI3K/Akt/mTOR | OSU-03012 |
| transmembrane transp. | amiodarone |
| angiogenesis | quizartinib |
| apoptosis | NSC319726 |
| others | AZ191 |
| ubiquitin | (-)-parthenolide |
| epigenetics | GSK J4 |
| protein tyrosine kinase | LDK378 |
| neuronal signaling | medetomidine |
| neuronal signaling | otilonium |
| cytoskeletal signaling | ispinesib |
| others | NH125 |
| ubiquitin | P22077 |
| JAK/STAT | stattic |
| ubiquitin | NSC697923 |
| neuronal signaling | solifenacin |
| neuronal signaling | ifenprodil |
| cytoskeletal signaling | SB743921 |
| protein tyrosine kinase | AZD3463 |
| ubiquitin | PR-619 |
| neuronal signaling | CTEP |
| angiogenesis | ibrutinib |
| apoptosis | pifithrin-µ |
| endocrinology & hormones | clomifene |
| neuronal signaling | JTC-801 |
| cell cycle | ehop-016 |
| stem cells & wnt | PF-5274857 |
| others | PFK15 |
| endocrinology & hormones | gestodene |
| GPCR & G protein | fingolimod |
| endocrinology & hormones | estradiol |
| JAK/STAT | HO-3867 |
| transmembrane transp. | lacidipine |
| ubiquitin | MNS |
| neuronal signaling | prucalopride |
| neuronal signaling | amitriptyline |
| protein tyrosine kinase | AZD9291 |
| neuronal signaling | ziprasidone |
| GPCR & G protein | BMS-833923 |
| cytoskeletal signaling | ARQ 621 |
| Neuronal signaling | escitalopram |
| endocrinology & hormones | altrenogest |
| TGF-beta/Smad | LDN-212854 |
| epigenetics | SRT1720 |
| ubiquitin | DBeQ |
| neuronal signaling | pergolide |
| GPCR & G protein | rimonabant |
| neuronal signaling | fluvoxamine |
| neuronal signaling | chlorpromazine |
| neuronal signaling | fluoxetine |
| neuronal signaling | clozapine |
| GPCR & G protein | NPS-2143 |
| neuronal signaling | duloxetine |
| neuronal signaling | dapoxetine |
| neuronal signaling | vortioxetine |
| neuronal signaling | chlorprothixene |
| neuronal signaling | paroxetine |
| cell cycle | CHIR-124 |
| JAK/STAT | SGI-1776 |
| - | - | ● | ●●● |
| --- | --- | --- | --- |
| ●● | ●● | ● | ●●● |
| - | ●● | - | ●●● |
| - | ●● | - | ●●● |
| - | ● | - | ●●● |
| - | - | ●● | ●● |
| - | - | - | ●●● |
| ● | ● | ●● | - |
| - | - | - | ●●● |
| - | ● | - | ●●● |
| ● | - | - | ●● |
| - | ●●● | - | ● |
| - | ●● | ● | ●●● |
| - | - | - | - |
| - | - | ●● | ●●● |
| | ● | - | - |
| - | - | - | ●●● |
| - | ●● | - | ●●● |
| - | ●● | - | ●●● |
| - | - | - | - |
| ● | - | - | - |
| - | ●● | ● | - |
| ● | ● | - | ● |
| - | ● | ●● | ●●● |
| - | - | - | ●●● |
| - | ● | ●●● | ●●● |
| - | - | ●● | - |
| - | - | - | - |
| - | ● | - | ●●● |
| - | ● | - | ●●● |
| ●● | - | - | ●●● |
| - | ● | - | - |
| | - | ●● | - |
| ● | - | ●●● | ●●● |
| - | ●●● | ● | - |
| - | ●●● | - | ●●● |
| ●●● | - | - | ●●● |
| | - | ● | ●● |
| ●● | ●● | ● | ●● |
| - | ●●● | ● | ●● |
| - | - | ●● | ●●● |
| ● | ●●● | - | - |
| ● | ●●● | ●●● | ● |
| - | ● | - | - |
| ● | ●● | ● | - |
| - | ●● | - | - |
| ●●● | ●●● | - | ● |
| - | ●●● | ●● | ●●● |
| - | ●●● | ●● | ●●● |
| - | ●●● | ● | ●●● |
| ●● | ● | - | - |
| ●●● | ●●● | - | ● |
| ● | ●●● | - | - |
| ● | ●●● | ● | ●● |
| - | ●●● | - | ●●● |
| ●● | ●●● | - | ●● |
| ● | ●●● | - | - |
| - | ●●● | - | ● |
| ●●● | ●●● | ●●● | ●●● |
| ●●● | ●●● | ●●● | ●●● |
| ●●● | ●●● | ● | ●● |
| ● | ●● | - | - |
| - | ●●● | - | ●●● |
| ●●● | ●●● | - | ●●● |
| - | ●●● | ●● | ●●● |
| ●●● | ●●● | - | - |
| ●●● | ●●● | ● | ●●● |
| ●●● | ●●● | ●●● | ●●● |
| ● | ●●● | - | - |
| - | ●●● | - | ●●● |
