# Supplementary material for: Identification of annotated bioactive molecules that impair motility of the blood fluke Schistosoma mansoni
Source: Int J Parasitol Drugs Drug Resist. 2020 Jun 1;13:73–88. doi: 10.1016/j.ijpddr.2020.05.002 (PMC7284125; doi:10.1016/j.ijpddr.2020.05.002)
Supplement: Multimedia component 2 [file mmc2.pptx]

## Slide 1
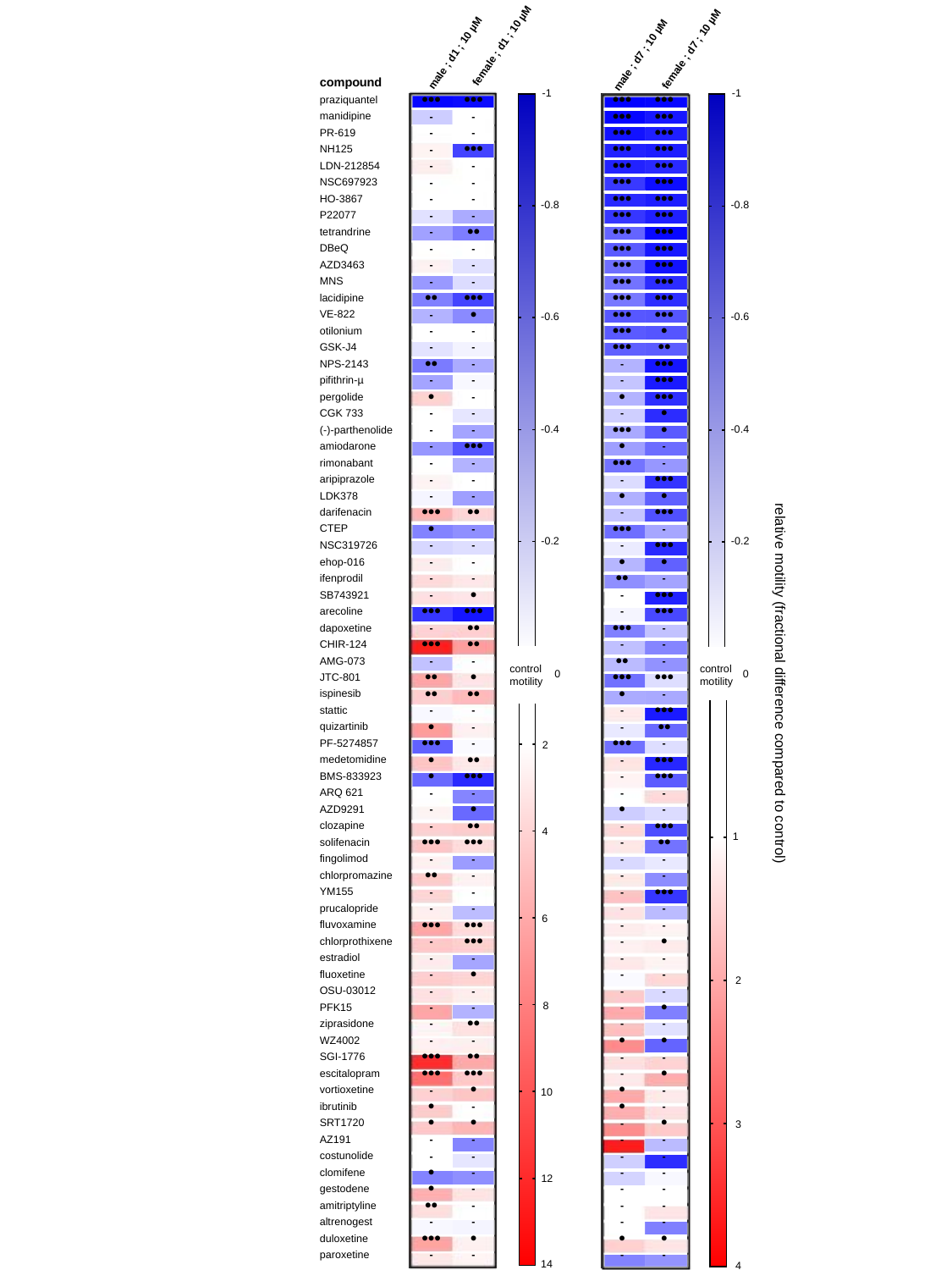

female ; d1 ; 10 µM
male ; d1 ; 10 µM
female ; d7 ; 10 µM
male ; d7 ; 10 µM
| compound |
| --- |
| praziquantel |
| manidipine |
| PR-619 |
| NH125 |
| LDN-212854 |
| NSC697923 |
| HO-3867 |
| P22077 |
| tetrandrine |
| DBeQ |
| AZD3463 |
| MNS |
| lacidipine |
| VE-822 |
| otilonium |
| GSK-J4 |
| NPS-2143 |
| pifithrin-µ |
| pergolide |
| CGK 733 |
| (-)-parthenolide |
| amiodarone |
| rimonabant |
| aripiprazole |
| LDK378 |
| darifenacin |
| CTEP |
| NSC319726 |
| ehop-016 |
| ifenprodil |
| SB743921 |
| arecoline |
| dapoxetine |
| CHIR-124 |
| AMG-073 |
| JTC-801 |
| ispinesib |
| stattic |
| quizartinib |
| PF-5274857 |
| medetomidine |
| BMS-833923 |
| ARQ 621 |
| AZD9291 |
| clozapine |
| solifenacin |
| fingolimod |
| chlorpromazine |
| YM155 |
| prucalopride |
| fluvoxamine |
| chlorprothixene |
| estradiol |
| fluoxetine |
| OSU-03012 |
| PFK15 |
| ziprasidone |
| WZ4002 |
| SGI-1776 |
| escitalopram |
| vortioxetine |
| ibrutinib |
| SRT1720 |
| AZ191 |
| costunolide |
| clomifene |
| gestodene |
| amitriptyline |
| altrenogest |
| duloxetine |
| paroxetine |
-1
-1
| ●●● | ●●● |
| --- | --- |
| - | - |
| - | - |
| - | ●●● |
| - | - |
| - | - |
| - | - |
| - | - |
| - | ●● |
| - | - |
| - | - |
| - | - |
| ●● | ●●● |
| - | ● |
| - | - |
| - | - |
| ●● | - |
| - | - |
| ● | - |
| - | - |
| - | - |
| - | ●●● |
| - | - |
| - | - |
| - | - |
| ●●● | ●● |
| ● | - |
| - | - |
| - | - |
| - | - |
| - | ● |
| ●●● | ●●● |
| - | ●● |
| ●●● | ●● |
| - | - |
| ●● | ● |
| ●● | ●● |
| - | - |
| ● | - |
| ●●● | - |
| ● | ●● |
| ● | ●●● |
| - | - |
| - | ● |
| - | ●● |
| ●●● | ●●● |
| - | - |
| ●● | - |
| - | - |
| - | - |
| ●●● | ●●● |
| - | ●●● |
| - | - |
| - | ● |
| - | - |
| - | - |
| - | ●● |
| - | - |
| ●●● | ●● |
| ●●● | ●●● |
| - | ● |
| ● | - |
| ● | ● |
| - | - |
| - | - |
| ● | - |
| ● | - |
| ●● | - |
| - | - |
| ●●● | ● |
| - | - |
| ●●● | ●●● |
| --- | --- |
| ●●● | ●●● |
| ●●● | ●●● |
| ●●● | ●●● |
| ●●● | ●●● |
| ●●● | ●●● |
| ●●● | ●●● |
| ●●● | ●●● |
| ●●● | ●●● |
| ●●● | ●●● |
| ●●● | ●●● |
| ●●● | ●●● |
| ●●● | ●●● |
| ●●● | ●●● |
| ●●● | ● |
| ●●● | ●● |
| - | ●●● |
| - | ●●● |
| ● | ●●● |
| - | ● |
| ●●● | ● |
| ● | - |
| ●●● | - |
| - | ●●● |
| ● | ● |
| - | ●●● |
| ●●● | - |
| - | ●●● |
| ● | ● |
| ●● | - |
| - | ●●● |
| - | ●●● |
| ●●● | - |
| - | - |
| ●● | - |
| ●●● | ●●● |
| ● | - |
| - | ●●● |
| - | ●● |
| ●●● | - |
| - | ●●● |
| - | ●●● |
| - | - |
| ● | - |
| - | ●●● |
| - | ●● |
| - | - |
| - | - |
| - | ●●● |
| - | - |
| - | - |
| - | ● |
| - | - |
| - | - |
| - | - |
| - | ● |
| - | - |
| ● | ● |
| - | - |
| - | ● |
| ● | - |
| ● | - |
| - | ● |
| - | - |
| - | - |
| - | - |
| - | - |
| - | - |
| - | - |
| ● | ● |
| - | - |
-0.8
-0.8
-0.6
-0.6
-0.4
-0.4
-0.2
-0.2
control
motility
control
motility
0
0
relative motility (fractional difference compared to control)
2
4
1
6
2
8
10
3
12
14
4
