# Supplementary material for: Identification of annotated bioactive molecules that impair motility of the blood fluke Schistosoma mansoni
Source: Int J Parasitol Drugs Drug Resist. 2020 Jun 1;13:73–88. doi: 10.1016/j.ijpddr.2020.05.002 (PMC7284125; doi:10.1016/j.ijpddr.2020.05.002)
Supplement: Multimedia component 3 [file mmc3.pptx]

## Slide 1
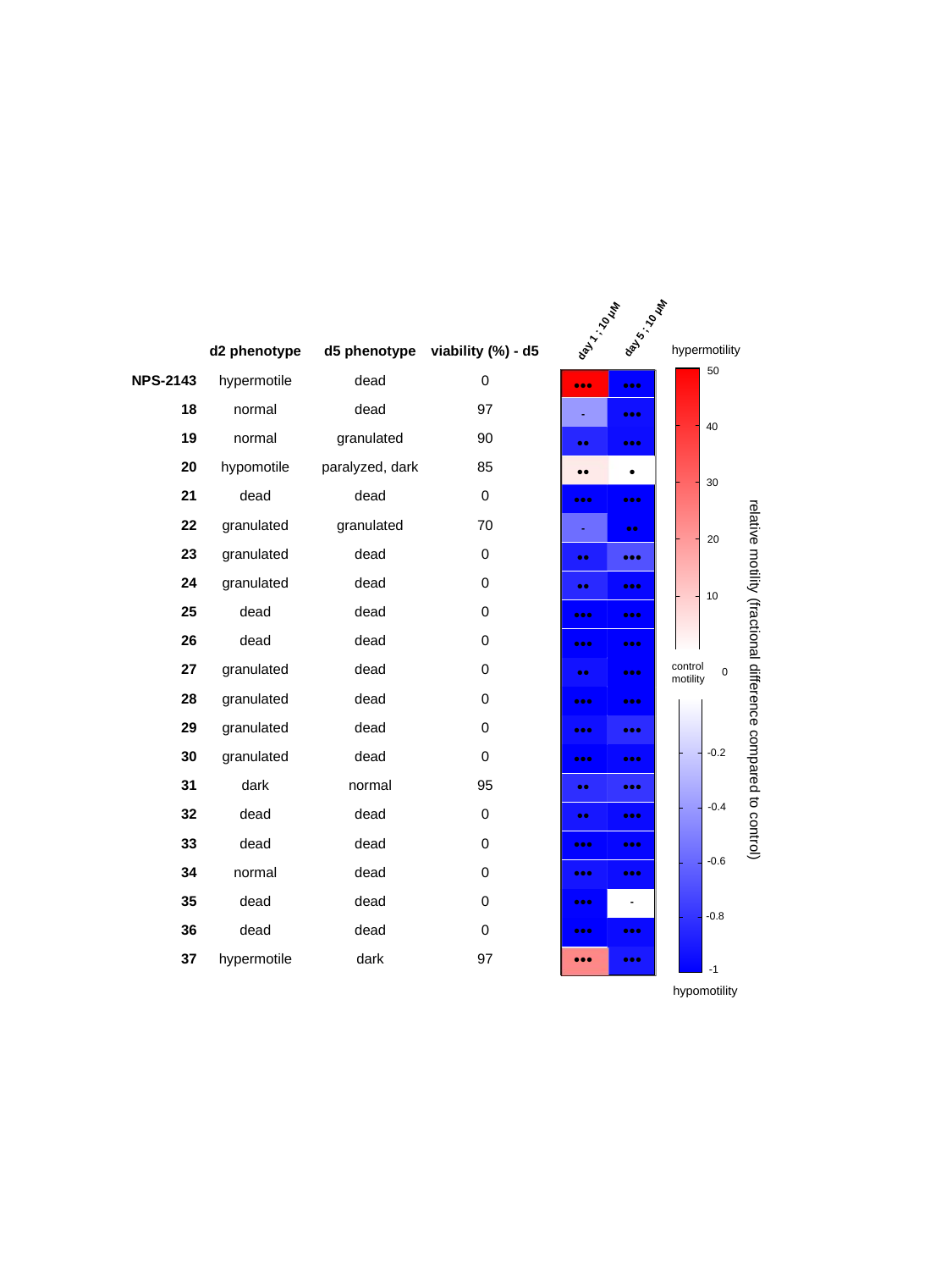

day 5 ; 10 μM
day 1 ; 10 μM
| | d2 phenotype | d5 phenotype | viability (%) - d5 |
| --- | --- | --- | --- |
| NPS-2143 | hypermotile | dead | 0 |
| 18 | normal | dead | 97 |
| 19 | normal | granulated | 90 |
| 20 | hypomotile | paralyzed, dark | 85 |
| 21 | dead | dead | 0 |
| 22 | granulated | granulated | 70 |
| 23 | granulated | dead | 0 |
| 24 | granulated | dead | 0 |
| 25 | dead | dead | 0 |
| 26 | dead | dead | 0 |
| 27 | granulated | dead | 0 |
| 28 | granulated | dead | 0 |
| 29 | granulated | dead | 0 |
| 30 | granulated | dead | 0 |
| 31 | dark | normal | 95 |
| 32 | dead | dead | 0 |
| 33 | dead | dead | 0 |
| 34 | normal | dead | 0 |
| 35 | dead | dead | 0 |
| 36 | dead | dead | 0 |
| 37 | hypermotile | dark | 97 |
hypermotility
50
| ●●● | ●●● |
| --- | --- |
| - | ●●● |
| ●● | ●●● |
| ●● | ● |
| ●●● | ●●● |
| - | ●● |
| ●● | ●●● |
| ●● | ●●● |
| ●●● | ●●● |
| ●●● | ●●● |
| ●● | ●●● |
| ●●● | ●●● |
| ●●● | ●●● |
| ●●● | ●●● |
| ●● | ●●● |
| ●● | ●●● |
| ●●● | ●●● |
| ●●● | ●●● |
| ●●● | - |
| ●●● | ●●● |
| ●●● | ●●● |
40
30
20
10
control motility
0
relative motility (fractional difference compared to control)
-0.2
-0.4
-0.6
-0.8
-1
hypomotility
