# Supplementary figures and images for: Identification of annotated bioactive molecules that impair motility of the blood fluke Schistosoma mansoni
Source: Int J Parasitol Drugs Drug Resist. 2020 Jun 1;13:73–88. doi: 10.1016/j.ijpddr.2020.05.002 (PMC7284125; doi:10.1016/j.ijpddr.2020.05.002)

## Slide 1
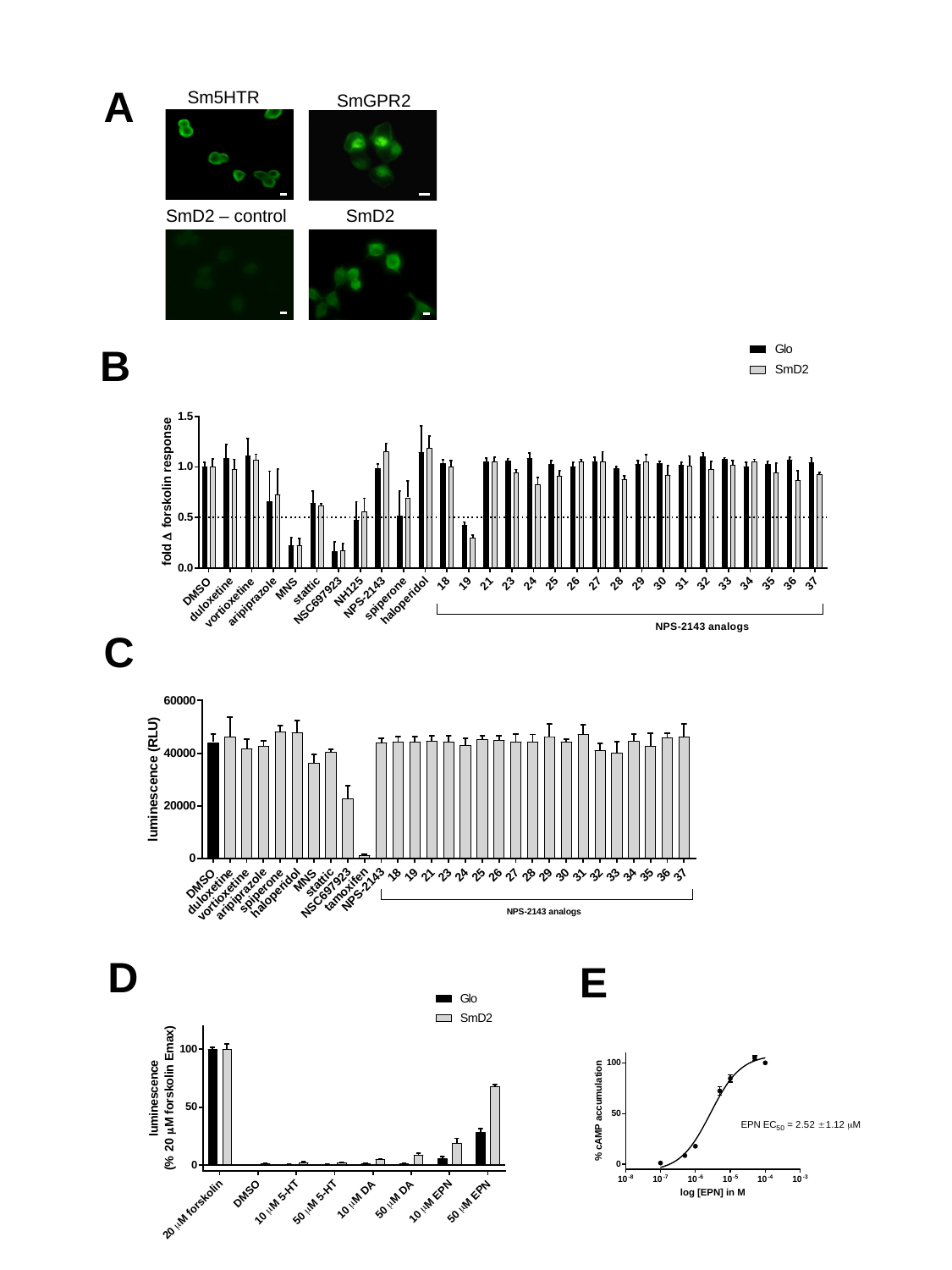

A
Sm5HTR
SmGPR2
SmD2
SmD2 – control
B
C
D
E

Supplement: Multimedia component 4 [file mmc4.pptx]
