# Supplementary material for: Identification of annotated bioactive molecules that impair motility of the blood fluke Schistosoma mansoni
Source: Int J Parasitol Drugs Drug Resist. 2020 Jun 1;13:73–88. doi: 10.1016/j.ijpddr.2020.05.002 (PMC7284125; doi:10.1016/j.ijpddr.2020.05.002)
Supplement: Multimedia component 10 [file mmc10.docx]

|  | Compounds | IC_50_ (μM) | S.E. |
| --- | --- | --- | --- |
|  | aripiprazole | 3.75 | 0.44 |
|  | duloxetine | 5.95 | 0.66 |
|  | vortioxetine | 3.68 | 0.43 |
|  | spiperone | 1.51 | 0.12 |
|  | haloperidol | 24.3 | 5.76 |
|  | NPS-2143 | 2.53 x 10^-3^ | 0.28 x 10^-3^ |
| NPS-2143 analogs | 18 | 0.22 | 0.03 |
|  | 19 | 2.01 | 0.31 |
|  | 23 | 2.71 | 0.7 |
|  | 24 | 1.35 | 0.21 |
|  | 25 | 0.49 | 0.08 |
|  | 26 | 3.33 | 0.64 |
|  | 27 | 1.96 | 0.24 |
|  | 28 | 27.24 x 10^-3^ | 3.79 x 10^-3^ |
|  | 30 | 0.34 | 0.08 |
|  | 31 | 21.70 x 10^-3^ | 2.31 x 10^-3^ |
|  | 32 | 0.17 | 0.05 |
|  | 33 | 0.18 | 0.05 |
|  | 34 | 9.36 | 3.34 |
|  | 35 | 12.26 x 10^-3^ | 1.53 x 10^-3^ |
|  | 36 | 5.41 | 2.15 |

***Supplementary Table 5: Relative affinity of SmD2 to hit compounds.***

*IC50s are represented in μM. S.E: standard error.*
